# Computational fluid dynamics model to predict the dynamical behavior of the cerebrospinal fluid through implementation of physiological boundary conditions

**DOI:** 10.3389/fbioe.2022.1040517

**Published:** 2022-11-22

**Authors:** Sarah Vandenbulcke, Tim De Pauw, Frank Dewaele, Joris Degroote, Patrick Segers

**Affiliations:** ^1^ Institute of Biomedical Engineering and Technology (IBiTech-bioMMeda), Department of Electronics and Information Systems, Ghent University, Ghent, Belgium; ^2^ Department of Neurosurgery, Ghent University Hospital, Ghent, Belgium; ^3^ Department of Electromechanical Systems and Metal Engineering, Ghent University, Ghent, Belgium

**Keywords:** cerebrospinal fluid, intracranial pressure, windkessel model, neurological disorders, computational fluid dynamics, cerebral blood vessels, cerebrospinal fluid absorption, intracranial compliance

## Abstract

Cerebrospinal fluid (CSF) dynamics play an important role in maintaining a stable central nervous system environment and are influenced by different physiological processes. Multiple studies have investigated these processes but the impact of each of them on CSF flow is not well understood. A deeper insight into the CSF dynamics and the processes impacting them is crucial to better understand neurological disorders such as hydrocephalus, Chiari malformation, and intracranial hypertension. This study presents a 3D computational fluid dynamics (CFD) model which incorporates physiological processes as boundary conditions. CSF production and pulsatile arterial and venous volume changes are implemented as inlet boundary conditions. At the outlets, 2-element windkessel models are imposed to simulate CSF compliance and absorption. The total compliance is first tuned using a 0D model to obtain physiological pressure pulsations. Then, simulation results are compared with *in vivo* flow measurements in the spinal subarachnoid space (SAS) and cerebral aqueduct, and intracranial pressure values reported in the literature. Finally, the impact of the distribution of and total compliance on CSF pressures and velocities is evaluated. Without respiration effects, compliance of 0.17 ml/mmHg yielded pressure pulsations with an amplitude of 5 mmHg and an average value within the physiological range of 7–15 mmHg. Also, model flow rates were found to be in good agreement with reported values. However, when adding respiration effects, similar pressure amplitudes required an increase of compliance value to 0.51 ml/mmHg, which is within the range of 0.4–1.2 ml/mmHg measured *in vivo*. Moreover, altering the distribution of compliance over the four different outlets impacted the local flow, including the flow through the foramen magnum. The contribution of compliance to each outlet was directly proportional to the outflow at that outlet. Meanwhile, the value of total compliance impacted intracranial pressure. In conclusion, a computational model of the CSF has been developed that can simulate CSF pressures and velocities by incorporating boundary conditions based on physiological processes. By tuning these boundary conditions, we were able to obtain CSF pressures and flows within the physiological range.

## 1 Introduction

Cerebrospinal fluid (CSF) flows freely around the brain and spinal cord in the central nervous system. This water-like fluid has multiple functions including mechanical protection of the neurological tissues and transport and secretion of brain metabolites and fluids (Spector et al., 2015; Bothwell et al., 2019). The dynamical behavior of CSF is the result of a complex interplay between different physiological mechanisms including CSF production and absorption, and interaction with the neurological tissues and the cardiovascular system (Bothwell et al., 2019; Matsumae et al., 2019). Non-physiological CSF pressures and velocities have been measured in patients with intracranial hypo- and hypertension (Johnston and Teo, 2000; Czosnyka et al., 2017), hydrocephalus (Bateman and Siddique, 2014), or Chiari malformation (Bunck et al., 2011). However, the exact processes altering the CSF dynamics and their relation with neurological complications are not well understood, and neither are the normal mechanisms governing CSF dynamics (Bothwell et al., 2019). A deeper understanding of CSF dynamics and the processes affecting them should advance our insight into CSF-related neurological disorders, which is critical to come up with better management and treatment of these disorders (Fillingham et al., 2022).

The CSF is not easily accessible for *in vivo* measurements and assessment of physiological processes and their impact on the CSF pressures and velocities is challenging. Pressure measurements with a ventricular catheter allow real-time CSF pressure monitoring but require invasive implementation of measurement devices and provide no spatial pressure data (Dai et al., 2020). In contrast, MRI imaging techniques including 4D flow MRI provide a non-invasive tool to study CSF flow *in vivo*. However, there is no real-time measurement (Baledent et al., 2006) and accurately capturing the slow CSF dynamics is not evident. Studies have reported important differences between scanner centers (Williams et al., 2021), artifacts, and limited MRI resolution (Heidari Pahlavian et al., 2016; Yavuz Ilik et al., 2022). Although these techniques provide valuable *in vivo* data, they provide little information on the processes impacting CSF dynamics and 4D flow only allows evaluation under specific conditions (in a supine position in an MRI machine).

Cadaver and animal studies are typically performed to investigate CSF turnover, whereby absorption should be equal to production to preserve stable CSF pressures. CSF is primarily produced by the choroid plexus in the cerebral ventricles, whereas multiple sites of absorption have been identified to drain CSF both into the venous and lymphatic system (Chen et al., 2015; Matsumae et al., 2016; Bothwell et al., 2019). CSF follows a pulsatile pattern corresponding to cardiac pulsations and breathing. This is because neurological tissues, cerebral blood, and CSF are encased by a bony skull and a less rigid spinal compartment (Kellie, 1824). Thus, when intracranial arteries increase in volume during systole, CSF is displaced within and out of the cranial compartment. The amount of CSF that is then moved into the spinal compartment depends on total arterial volume change and compliance of the spinal and cranial compartments. It has been estimated that, in a lying position, the spinal compartment contributes 63%–73% to compliance in CSF buffering, but posture and changes in physiology can change these values (Magnaes, 1989; Tain et al., 2011). Besides arterial pulsations, also respiration impacts CSF motion. Yamada et al. (2013) used a non-invasive spin labeling technique to measure CSF motion in a person and observed significant movement of CSF during deep respiration with CSF flowing rostral (upward) during inspiration and caudal (downward) during expiration. These movements are the result of volume changes in cerebral veins caused by changes in intrathoracic pressure during breathing (Aktas et al., 2019).

Computational fluid dynamics (CFD) models allow for a detailed evaluation of fluid pressures and velocities within complex fluid spaces under different (patho)physiological conditions. Because of the complexity of the CSF space, most CFD studies focused on a small part of the CSF geometry (Gupta et al., 2009; Clarke et al., 2013; Heidari Pahlavian et al., 2016; Sass et al., 2017). This allowed studying local effects but did not provide insight into the system-wide responses. Increasing computational resources and advances in medical imaging, have enabled research of the highly complex CSF flow. Recently, Khani et al. (2020) developed a CFD model of the full CSF circulation thereby including constant CSF production in the lateral ventricles, CSF pulsations at the caudal end of the spinal compartment as measured at the cervical (vertebra C2-C3) level, and a zero-pressure outlet at the cranial opening. As this model was intended to compare blood removal from the CSF between two filtration systems, i.e. lumbar drain and a dual-lumen catheter-based CSF filtration system, a drainage rate corresponding to the filtration system was specified and a multiphase model was used to incorporate the presence of blood in the CSF. In a follow-up study, this model was expanded by adding a respiration pulsation term at the caudal end (Khani et al., 2022). Fillingham et al. (2022) conducted CFD simulations with boundary conditions directly based on phase-contrast MRI measurements of the CSF and cardiac in- and outflow with the aim to capture CSF flow more realistically (Fillingham et al., 2022). These studies did model pressure differences, but no absolute pressures as recorded during intracranial pressure monitoring, which requires taking compliance and absorption resistance into account. In contrast, 0D models have been successfully developed to model intracranial pressure dynamics and compliance (Wakeland and Goldstein, 2008). In 1975, Marmarou modeled CSF pressure dynamics assuming a single CSF compartment, venous absorption, and constant CSF formation by using an electrical analog (Marmarou et al., 1975). In the next decades, models became gradually more complex by coupling CSF with cerebral arterial and venous blood flow (Hoffmann, 1987; Ursino, 1988; Czosnyka et al., 1997). Interestingly, (Tain et al., 2011), implemented windkessel models in a 0D model to estimate heterogenous CSF compliance based on phase-contrast MRI. Although these models provided valuable information related to intracranial pressure and compliance, they do not account for spatial differences in flow and pressure within the CSF space. To the best of our knowledge, only one study included spinal compliance in a patient-based 3D computational model of the CSF. In that work, spinal compliance was implemented through a fluid-structure interaction (FSI) interface corresponding to the dura mater (Sweetman et al., 2011).

While the aforementioned studies provided valuable insights and results, CFD studies only presented spatial pressure differences and thus provided no absolute pressures over time as recorded through intracranial pressure measurements. Also, although there is evidence for fluid exchange with the interstitium and lymphatic absorption (Brinker et al., 2014; Matsumae et al., 2019), only venous absorption through the arachnoid villi at the top of the cranial subarachnoid space (SAS) was considered. CFD models did not account for the impact of CSF compliance, and no 3D computational studies were found that incorporate the compliance distribution over the cranial and spinal compartments.

This work aims to build a robust computational model, which can realistically predict CSF pressures and velocities under different physiological conditions. Therefore, a three-dimensional CFD model was developed that allows the simulation of absolute pressures and velocities within the complex CSF space, which are validated against *in vivo* measurements of pressure and flow reported in the literature. We address some limitations of previous studies through the implementation of CSF production, secretion into the lymphatic and venous system, arterial and venous volume changes, and intracranial and spinal compliance.

## 2 Materials and methods

A CFD model of the CSF circulation was constructed from medical images and literature data following four steps: 1) Segmentation of the 3D geometry, 2) generation of computational mesh, 3) implementation of boundary conditions, and 4) set up of numerical solver. These are elucidated in detail in the next paragraphs. The CFD analysis was performed using the numerical software Fluent 2021 R2 (Ansys, Canonsburg, United States).

### 2.1 Model geometry

**FIGURE 1:**
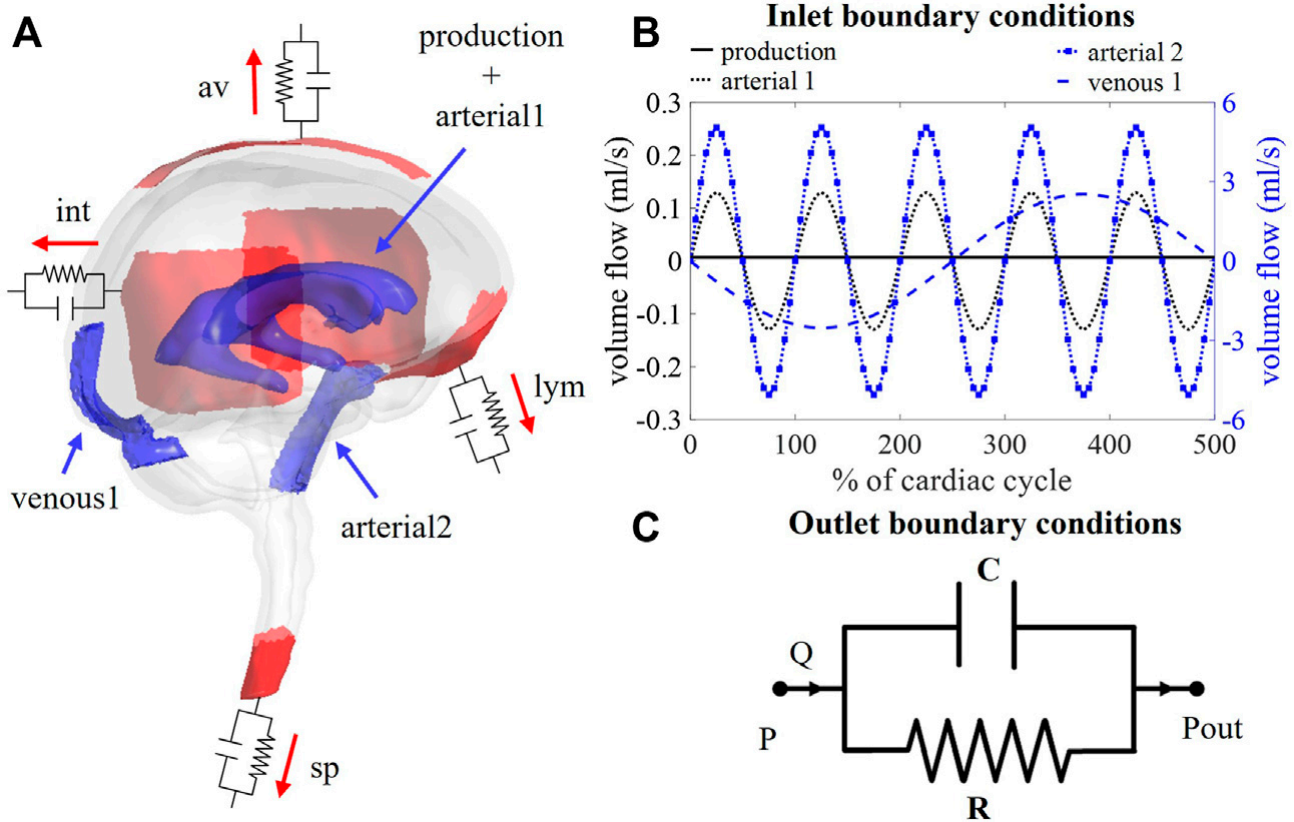
**(A)** Visualization of boundary conditions in the 3D model. Production and arterial1 designate the surface of the lateral ventricles (in blue), while arterial2 and venous1 point at the CSF volumes around the basilar artery and the cervical cerebral veins, respectively (in blue). The four outlet boundary conditions are designated in red and correspond to the interstitium (int), spinal (sp), lymphatic (lym), and arachnoid villi (av) outlet. **(B)** Graph containing the waveforms of the four different inlet boundary conditions depicted in **(A)**. **(C)** Electrical circuit representing the 2-element windkessel model, which is imposed at the outlets, containing a resistance R and compliance C in parallel. A pressure difference P–P_out_ is created when a flow Q passes through the circuit.

First, the three-dimensional geometry was segmented from clinical T2 MRI images of a patient with Chiari type 1 malformation (Figure 1A) using Mimics 21.0 (Materialise, Leuven). These image data were collected at Ghent university hospital using a 3 T Prisma system (Siemens, München, Germany) and exist of sagittal 2D slices (in-plane resolution of 0.625 × 0.625 mm) of 3 mm. The use of the anonymized data for this study was approved by the ethical committee of Ghent University Hospital. The geometry of the cranial CSF space is complex, especially because of the seepage of CSF between the folds of the brain surface. These irregularities can significantly increase the necessary computational effort if they would have been included in the model. For that reason, some simplifications of the geometry were performed: a uniform thickness was assigned to the cranial SAS and the complete geometry was smoothed using Mimics 24.0 and 3-Matic 16.0 (Materialise, Leuven). It is important to note that these modifications cleared the blockage of CSF flow characterizing Chiari type 1 malformation with a SAS thickness of minimally 4 mm at the level of the foramen magnum in the 3D model.

### 2.2 Computational mesh

A computational mesh was generated using ICEM 2021 R2 (Ansys, Canonsburg, United States). The mesh is composed of tetrahedral and prismatic elements: tetrahedra occupy the largest part of the volume and three prism layers with a total thickness of 0.7 mm were created next to the walls to accurately resolve the laminar velocity profile. The tetrahedral mesh elements have a maximal seed size of 3 mm and a refinement factor of 10 was applied. The thickness of each prism layer is determined by an exponential growth law with height ratio 2 and the total thickness. The filet ratio, maximal prism angle, maximal height over base, and prism height limit factor were set to 1, 180, 0.8, and 1 respectively. A mesh sensitivity study was executed for a stationary flow case with a constant velocity inlet of 0.4 ml/min and zero pressure boundary condition at the interstitial outlet and outflow boundaries for the spinal, lymphatic, and arachnoid villi outlets with 20%, 30%, and 30% of outflow, respectively. Spinal SAS pressure, wall shear stress, maximal velocity, and average pressure were evaluated for 13 meshes ranging from 0.3 to 5 million cells. Based on this study, the mesh with 1.18 million cells was selected which is the coarsest mesh with a maximal deviation of 5% compared to the finest mesh.

### 2.3 Boundary conditions

Boundary conditions are based on our current understanding of physiological processes impacting the CSF flow. An overview of the applied in -and outlet boundary conditions is depicted in Figure 1A.

#### 2.3.1 Inlet boundary conditions

##### 2.3.1.1 CSF production

First, at the lateral ventricles (see Figure 1A, zones indicated in blue), a constant velocity inlet of 5.57E-04 mm/s was imposed over a surface of 11,955 mm^2^ to account for constant CSF production of 0.4 ml/min (Rubin et al., 1966; Brinker et al., 2014). The corresponding flow rate (Q_production_) is presented as production in Figure 1B; Table 1.

**TABLE 1:** Overview of average values (avg.) and amplitudes (amp.) of all the inlet boundary conditions.

Name	Area	Type	Avg. (ml/s)	Amp. (ml/s)
**production**	Lateral ventricles	Constant	6.67E-3	—
**arterial1**	Lateral ventricles	Sine wave 1 Hz	—	0.11
**arterial2**	Basilar region	Sine wave 1 Hz	—	5.05
**venous1**	Cerebral veins occipital	Sine wave 0.2 Hz	—	1.01

##### 2.3.1.2 Cardiac pulsations

Second, cerebral arteries undergo volume changes along the cardiac cycle and small arteries lying inside the parenchyma induce a pulsatile motion of the brain tissue lining the cerebral ventricles (Matsumae et al., 2019). This effect has been accounted for by adding a sinusoidal velocity waveform with a frequency of 1 Hz and zero net flow to the constant CSF production. Following the conservation of mass, the flow amplitude of the sinusoidal signal (Q_arterial1_) is derived from volumetric flow measurements at the level of the 3^rd^ ventricle (Q_v3_) (Sweetman et al., 2011). A value of 0.11 ml/s is selected and depicted as arterial1 in Figure 1B; Table 1.

$$Q_{Arterial1\;}={\;Q}_{v3}=0.11\;ml/s$$ (1)

Volume changes of large arteries including the basilar artery result in important CSF displacements. These volume changes are implemented by adding a sinusoidal mass source term in the basilar artery region of the CSF (volume 9.53 ml). The amplitude of the corresponding volumetric flow Q_arterial2_ caused by these volume changes is estimated from cervical (Q_c_) and aqueduct (Q_v3_) CSF flow measurements and the relative contribution of the spinal compartment to the total CSF compliance (c_sp_; see also section 2.3.2.2).

$$Q_{arterial2}=\frac{{\;Q}_{c}-Q_{v3\;}}{c_{sp}}$$ (2)

Because important differences between cervical measurements reported in literature exist (range varying from 1.5 to 6 ml/s (Tangen et al., 2015; Benninghaus et al., 2019; Fillingham et al., 2022; Khani et al., 2022)), an average value of 5.06 ml/s was chosen for Q_arterial2_ (arterial2 in Figure 1B; Table 1).

##### 2.3.1.3 Respiratory effects

Recent studies have measured CSF displacements at the cervical level in response to respiration (Yamada et al., 2013; Yildiz et al., 2017). These are caused by volume changes of veins that interact with the CSF. Therefore, venous volume changes are added as a pulsating 0.2 Hz source term with zero net flow in the fluid zone corresponding to the occipital cranial veins (venous1 in Figures 1A,B; Table 1). The amplitude of the respiratory pulsations (Q_venous1_) was considered 50% of the amplitude of the arterial pulsations based on cervical flow measurements by (Yildiz et al., 2017).

#### 2.3.2 Outlet boundary conditions

The model consists of four outlets corresponding to the different CSF absorption pathways into the venous and lymphatic system: interstitial (int), spinal (sp), lymphatic (lym), and arachnoid villi (av) absorption pathway (Chen et al., 2015; Matsumae et al., 2016; Bothwell et al., 2019). To account for both absorption resistance and CSF compliance, windkessel boundary conditions are imposed at each outlet. In particular, the 2-element windkessel model is applied corresponding with an electrical analog containing resistance (R) and compliance (C) in parallel (Westerhof et al., 2009) as shown in Figure 1C. Pressure (P) and flow (Q) at each outlet are then related by

$$Q=\frac{P-P_{out}}{R}+C\frac{d\left(P-P_{out}\right)}{dt}$$ (3)

where P_out_ corresponds to the external pressure which is in all cases set to zero (Xiao et al., 2014).

##### 2.3.2.1 Windkessel boundary conditions: Implementation in CFD solver

Resistance and compliance are accounted for by coupling a 2-element windkessel to each outlet of the CFD model. Therefore, the pressure at each outlet i at timestep n is set following the differential equation coupling pressure and flow (Eq. 3).

$$Q_{i,n}=\frac{P_{i,n}}{R_{i}}+C_{i}\frac{dP_{i,n}}{dt}$$ (4)

Because Fluent is a black-box solver, this differential equation cannot be solved simultaneously with the flow equations. Therefore, an explicit expression is derived by discretizing Eq. 4 over time.

$$\left\{ \begin{array}{c} dt=∆t\\ {dP}_{i,n}=P_{i,n}-P_{i,n-1} \end{array}\right.$$ (5)

where ∆t is the time step size, and P_i,n_ and P_i,n-1_ pressure in the current and previous step, respectively. This results in an expression of the pressure at outlet i in function of the outflow at timestep n and the pressure in the previous timestep n-1

$$P_{i,\;n}=\frac{Q_{i,\;n}R_{i}+P_{i,\;n-1}\frac{C_{i}R_{i}}{∆t}}{1+\frac{C_{i}R_{i}}{∆t}}$$ (6)

where C_i_ is the compliance and R_i_ the resistance at outlet i. To couple this equation to the CFD solver for all outlets, the algorithm described in (Annerel et al., 2010), developed in the context of a fluid-structure interaction (FSI) problem, was adapted to control the interaction between the outlets with the pressure and flow governed by the windkessel formulation (Eq. 4). The coupling first determines the linearized relation between the flow rates and pressures at all outlets and then includes this linearized model in the boundary conditions. This is to enable a strong implicit coupling of the pressure at the outlets and the fluid flow and overcome convergence issues that we experienced with an explicit coupling scheme, ascribed to the very small spatial pressure differences (order 0.01 mmHg) compared to large pressure differences over time (order 10 mmHg). Further details on the coupling algorithm are found in the supplementary material.

##### 2.3.2.2 Resistance values

R_tot_ is calculated as the ratio of average intracranial pressure (ICP_avg_) and the CSF production rate (Q_production_). The average intracranial pressure is assumed 10 mmHg within the normal physiological range of 7–15 mmHg (Eide and Kerty, 2011; Dai et al., 2020; Singh and Cheng, 2021).

$$R_{tot}=\frac{{ICP}_{avg}}{Q_{production}}=\frac{10\;mmHg}{0.00667\;ml/s}≅1500\;mmHg.s/ml$$ (7)

The four outlet resistances (R_i_) are placed in parallel, contributing to total resistance as

$$R_{tot}=\frac{1}{\frac{1}{R_{\mathrm{int}}}+\frac{1}{R_{sp}}+\frac{1}{R_{lym}}+\frac{1}{R_{av}}}\;with\;R_{i}=\frac{R_{tot}}{q_{i}}$$ (8)

With R_int_, R_sp_, R_lym_, and R_av_ the interstitial, spinal, lymphatic, and arachnoid villi outlet resistance, and q_i_ is the percentage of the total net outflow passing through the outlet i. The exact distribution of absorption *via* each of these outlets is debated. Only in recent years CSF absorption along other pathways than the arachnoid villi were identified, and studies reported lymphatic absorption ranging from 0% to 50% of the total CSF uptake (Brinker et al., 2014; Chen et al., 2015). In this study, 30% outflow is assigned to both the lymphatic and arachnoid villi outlet. An overview of the resistance values is provided in Table 2.

**TABLE 2:** Overview of resistance values at the four different outlets.

	Net outflow (%)	Resistance (mmHg.s/ml)
Outlet int	20	7,500
Outlet sp	20	7,500
Outlet lym	30	10,000
Outlet av	30	3,000
Total	100	1,500

##### 2.3.2.3 Compliance values

The total compliance in the model is the sum of the compliances of each outlet (C_i_), corresponding to capacitors placed in parallel.

$$C_{tot}=C_{\mathrm{int}}+C_{sp}+C_{lym}+C_{av}\;with\;C_{i}=c_{i}C_{tot}$$ (9)

**FIGURE 2:**
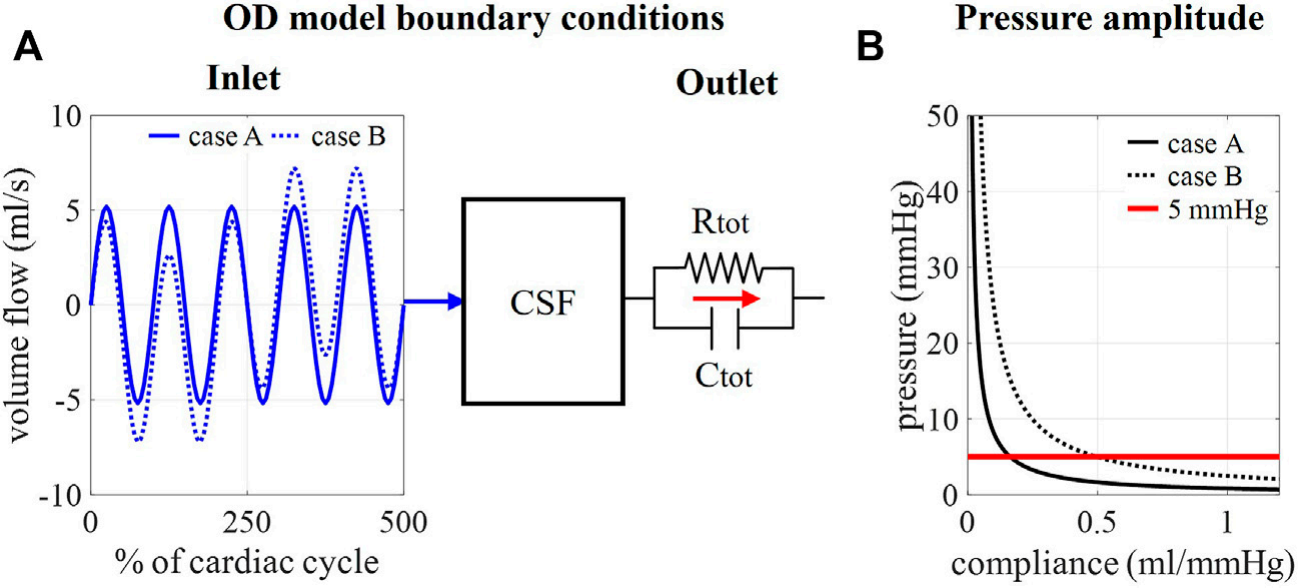
**(A)** Schematic of the 0D model assuming one inlet and one 2-element windkessel outlet. **(B)** Fitting compliance values for cases A and B using the 0D model. Pressure pulsation amplitudes for 1,000 compliance values ranging from 0 to 1.2 ml/mmHg (black curves) are visualized together with the targeted amplitude of 5 mmHg (red curve).

Here, c_i_ is the contribution of outlet i (in %) to total compliance. For the spinal outlet, this value is initially set to 66% based on *in vivo* reports with values ranging from 63% to 73% spinal compliance contribution for humans in lying position (Magnaes, 1989; Tain et al., 2011). The total compliance is estimated by calculating the difference between maximal and minimal pressure over five cardiac cycles for compliance values ranging from 0 to 1.2 ml/mmHg. These compliance values are based on *in vivo* CSF compliance between 0.4 and 1.2 ml/mmHg as reported by (Magnaes, 1989; Portella et al., 2005; Tain et al., 2011; Eide, 2016). To avoid the need for a large number of 3D CFD simulations, a 0D model is used to estimate the 5-s interval peak-to-peak pressure difference for multiple values of total compliance. Figure 2A shows a schematic of this 0D model. In this 0D model, the intracranial pressure is approximated assuming one inlet that combines all inflow boundary conditions, Q_in_.

$$Q_{in}=Q_{production}+Q_{arterial1-2}\sin\left(2\pi t\right)+Q_{venous1}\left(t\right)\sin\left(2\pi tf_{venous1}\right)$$ (10)

The discretized expression (Eq. 6) is then adapted for one outlet to calculate pressure (P_n_) at time step n

$$P_{n}=\frac{Q_{n}R_{tot}+P_{n-1}\frac{C_{tot}R_{tot}}{∆t}}{1+\frac{C_{tot}R_{tot}}{∆t}}$$ (11)

With P_n-1_ the pressure in the previous timestep and timestep size ∆t set equal to 0.05 s. The compliance value C_tot_ that leads to intracranial pressure pulsations with an amplitude of 5 mmHg is then selected. This approach is applied for the calculation of compliance for cases A (Q_venous1_ is zero) and B.

#### 2.3.3 Validation and boundary condition analysis

Five different cases are set up to validate the CFD simulation outcomes against reported literature data and investigate the impact of respiration and compliance distribution on CFD simulation outcomes.

In **case A**, only production and cardiac pulsations are accounted for as inlet conditions, thus discarding the impact of respiration. Outlet boundary conditions are resistances for the arachnoid villi (av) and lymphatic (lym) outlet, and 2-element windkessel models for the interstitial (int) and spinal (sp) outlet with compliance distribution of 33 and 66% respectively. The total compliance is calculated following section 2.3.2.3. In **case B**, respiratory effects are added to the inlet boundary conditions described in case A, while outlet boundary conditions do not change. This addition leads to a new value of total compliance, again calculated following subsection 2.3.2.3. In **case C**, inlet boundary conditions described in case B are imposed, while outlet boundary conditions are 2-element windkessel models for all outlets with compliance distribution of 11%, 66%, 11%, and 11% for the interstitial (int), spinal (sp), lymphatic (lym), and arachnoid villi (av) outlet respectively. The total compliance is unchanged compared to case B. In **case D**, the spinal and cranial compliance are reversed compared to case B. Hence, outlet boundary conditions are resistances for the arachnoid villi (av) and lymphatic (lym) outlet, and 2-element windkessel models for interstitial (int) and spinal (sp) outlet with compliance distribution of 66% and 33% respectively. Total compliance is the same as in cases B and C. Finally, in **case E**, inlet and outlet boundary conditions are the same as in case B but the total compliance is doubled.

Table 3 shows an overview of the inlet conditions, compliance distribution, and total compliance. Only simulation results of case A are compared with phase-contrast MRI measurements from the literature. This is because these measurements are cardiac-gated. Results of all cases as compared with pressure values from the literature.

**TABLE 3:** Overview of inlet conditions with average (avg.) and amplitude (amp.) and distribution of and value of compliance for cases A, B, C, D, and E.

		Case A	Case B	Case C	Case D	Case E
Inlet conditions (ml)	Production avg	6.67E-3	6.67E-3	6.67E-3	6.67E-3	6.67E-3
	Production amp	—	—	—	—	—
	Arterial1 avg	—	—	—	—	—
	Arterial1 amp	0.11	0.11	0.11	0.11	0.11
	Arterial2 avg	—	—	—	—	—
	Arterial2 amp	5.05	5.05	5.05	5.05	5.05
	Venous1 avg	—	—	—	—	—
	Venous1 amp	—	1.01	1.01	1.01	1.01
Compliance Distribution (%)	int (cint)	33	33	11	67	33
	sp (csp)	67	67	67	33	67
	lym (clym)	—	—	11	—	—
	av (cav)	—	—	11	—	—
Compliance (ml/mmHg)	Total Ctot	0.17	0.51	0.51	0.51	1.01

### 2.4 Solver settings

The CSF was modeled as an incompressible Newtonian fluid with properties the same as water (density 998.2 kg/m³ and dynamic viscosity of 0.001003 kg/m.s). The motion of the fluid was governed by the continuity and Navier-Stokes equations.

$$\begin{array}{c} \nabla ∙u=0\\ \rho \frac{\partial u}{\partial t}+\rho u∙\nabla u=-\nabla p+\mu \nabla^{2}u \end{array}$$ (12)

With the fluid density ρ [kg/m³], the velocity vector u [m/s], the fluid viscosity μ [kg/m.s], and the pressure field p [Pa]. The flow is considered incompressible, the effects of gravity were neglected, and the flow was considered laminar because of low Reynolds numbers, i.e., maximal order of 100s (35 and 341 at the level of the cerebral aqueduct and cervical SAS respectively). The transient simulations were run using a PISO scheme and a finite volume method with 2^nd^ order spatial and temporal discretization. An absolute convergence criterion of 1E-9 was imposed for all residuals (velocity in 3 spatial directions, pressure). All simulations were run using a time step of 0.05 s. The central computing infrastructure of Ghent university (HPC) was used for the simulations using up to 96 processor cores. Simulation of 25 cardiac cycles or a total simulation time of 25 s took about 14 h to complete.

## 3 Results

### 3.1 Tuning total compliance using a 0D model

**FIGURE 3:**
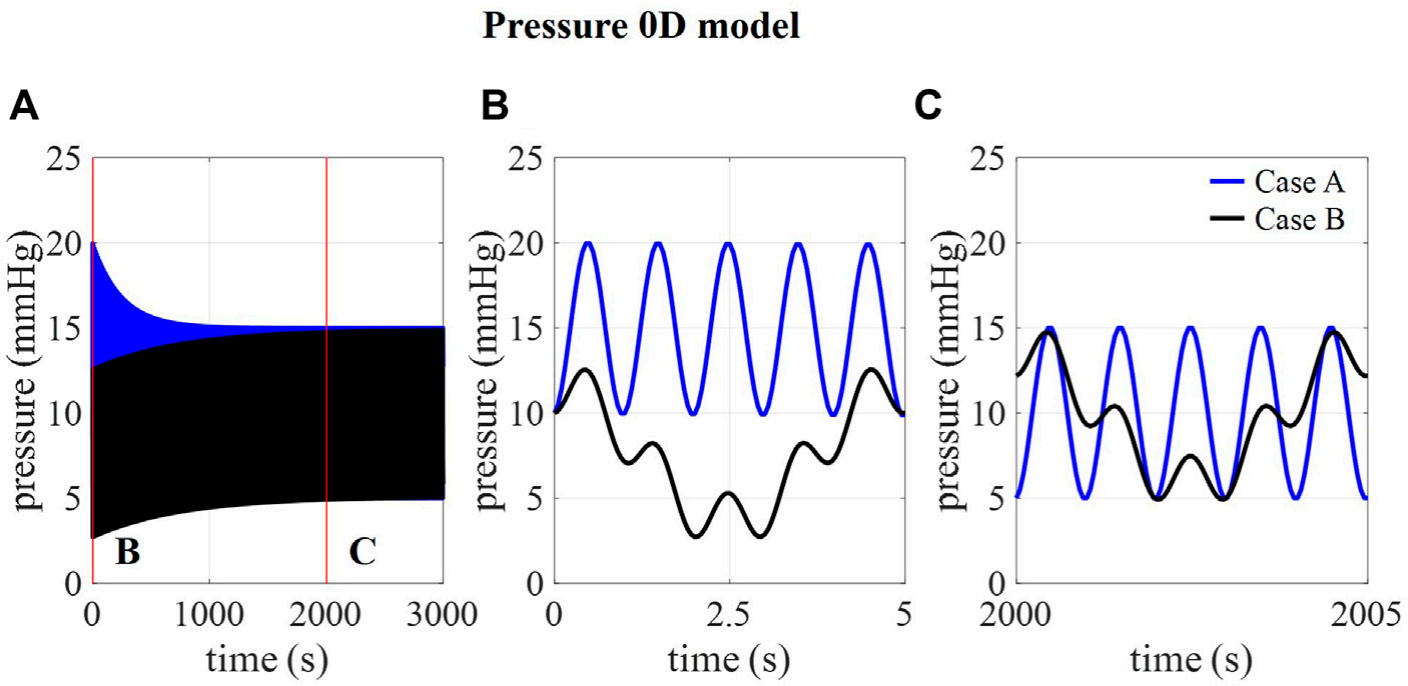
. **(A)** 0D prediction of pressure over 3,000 cardiac cycles for cases A and B and detail of pressure at **(B)** 0–5 s and **(C)** 2000–2005 s.

In Figure 2B, the amplitudes of pressure pulsations are shown for 1,000 compliance values ranging from 0 to 1.2 ml/mmHg as calculated using the 0D approximation. The cross-section of the two curves with the targeted amplitude of 5 mmHg corresponds with a compliance value of 0.17 and 0.51 ml/mmHg for case A and B, respectively. With these compliance values, the intracranial pressure is calculated using the 0D model for 3,000 cardiac cycles (Figure 3A) showing a transient phenomenon over time.

In the first five cardiac cycles (Figure 3B), the average pressure is 14.9 mmHg for case A compared to 7.7 mmHg for case B. In contrast, after 2000 cardiac cycles, average pressure approaches the targeted average intracranial pressure for both cases (10.0 mmHg for case A and 9.9 mmHg for case B). In the next paragraphs, CFD simulation results are obtained using 0.17 ml/mmHg for case A, 0.51 for cases B, C, and D, and 1.01 ml/mmHg for case E as presented in Table 3.

### 3.2 Validation against *in vivo* CSF flow measurements (case A)

**FIGURE 4:**
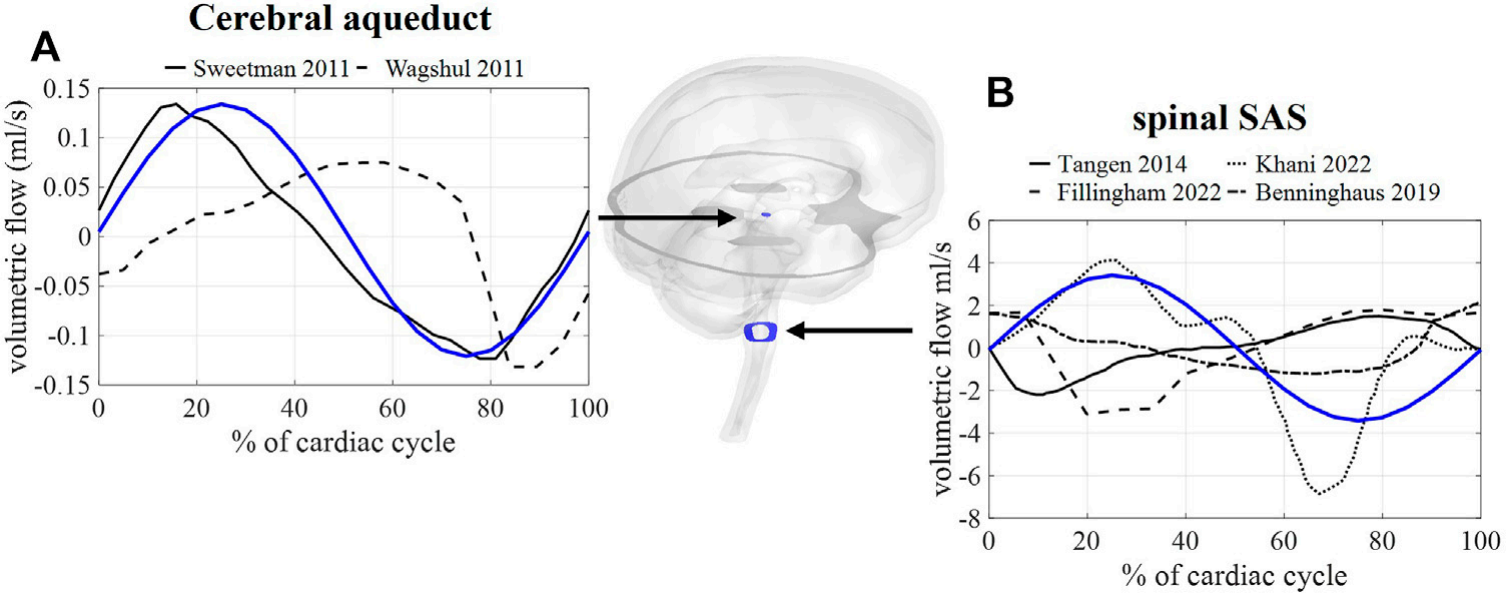
CFD simulation results (blue) and literature measurements (black) of CSF flow through 2 cross-sections: **(A)** flow through a cross-section of the cerebral aqueduct with literature values reported in and reproduced from (Sweetman et al., 2011, Wagshul et al.). **(B)** Flow through spinal SAS space cross-section with literature values reported in and reproduced from (Tangen et al., 2015; Benninghaus et al., 2019; Fillingham et al., 2022; Khani et al., 2022).

First, we consider the results corresponding with case A, thus without the inclusion of respiration. In Figure 4, the calculated and measured CSF flow are visualized for two locations: the spinal SAS and the cerebral aqueduct. The amplitude of the pulsations in the cerebral aqueduct is both *in vivo* and *in silico* in the order of 0.1 ml/s whereas pulsations through the foramen magnum are more than 10 times larger in the range of 2–5 ml/s.

### 3.3 Impact of respiration on CSF dynamics (Case B)

#### 3.3.1 CSF flow

**FIGURE 5:**
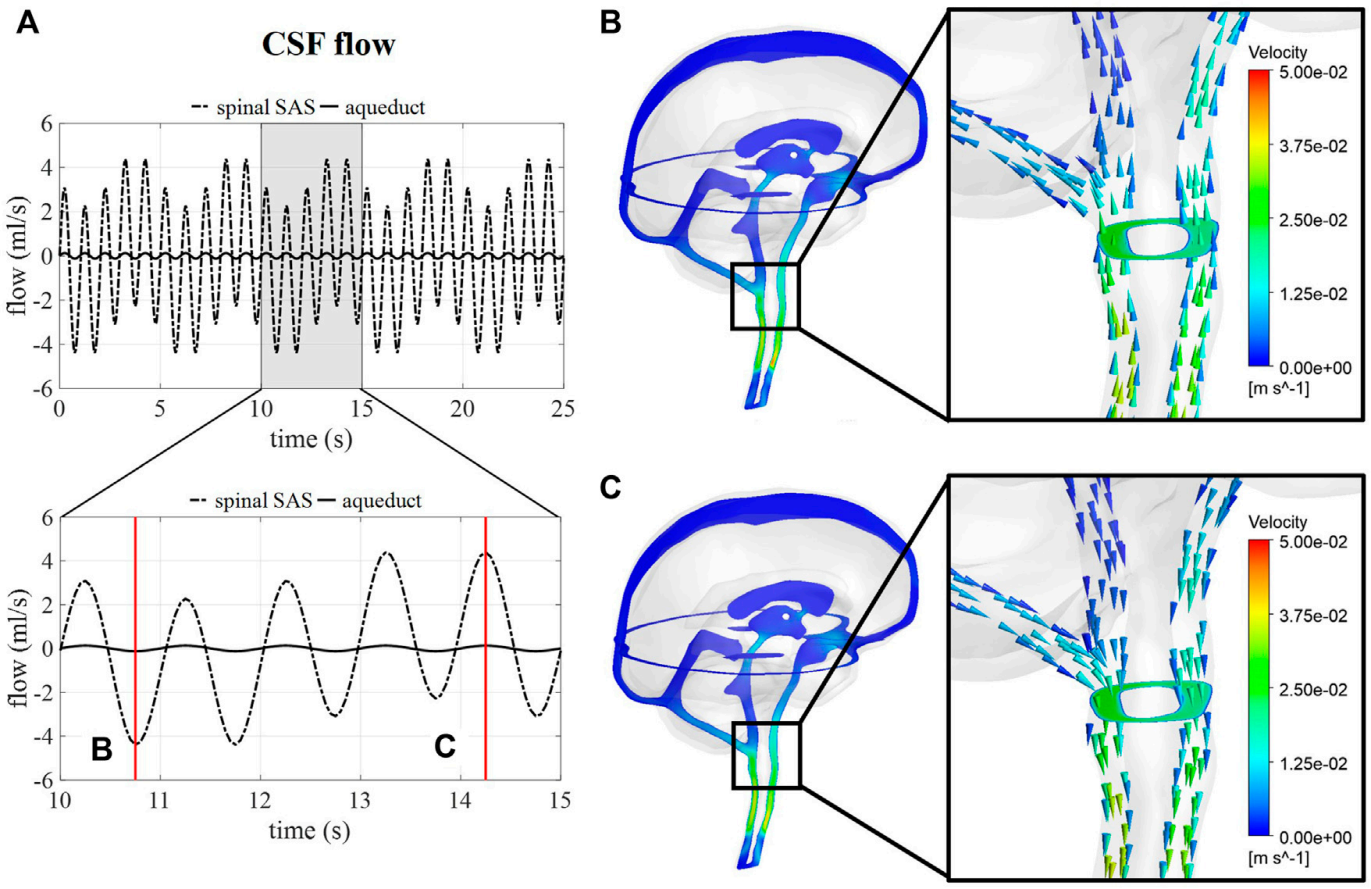
CSF flow and velocity simulations for case **(B) (A)** CSF flow through a cross-section of the cerebral aqueduct and the spinal SAS for 25 s and then zoomed in for 5 s. **(B)** and **(C)** CSF velocities after 10.75 and 14.25 s, respectively.

Figure 5A shows the flow through a cross-section of the cerebral aqueduct and the spinal SAS simulated for 5 respiratory cycles and 25 cardiac cycles corresponding with case B. The inclusion of pulsations at the level of the ventricles and in the basilar region is responsible for the fast pulsations of 1 Hz found in the cervical region, whereas slower fluctuations are attributed to venous volume changes implemented at the cranial veins. The amplitude of the pulsations at the cross-section of the SAS is not only proportional to applied venous and arterial pulsations but also to the 66% spinal compliance contribution. In Figures 5B,C the CSF velocities are visualized for 2 time points showing a clear direction switch of the velocity vectors along the respiratory cycle. Flow velocity through the cerebral aqueduct is maximally about 1.2 cm/s.

#### 3.3.2 CSF pressure

**FIGURE 6:**
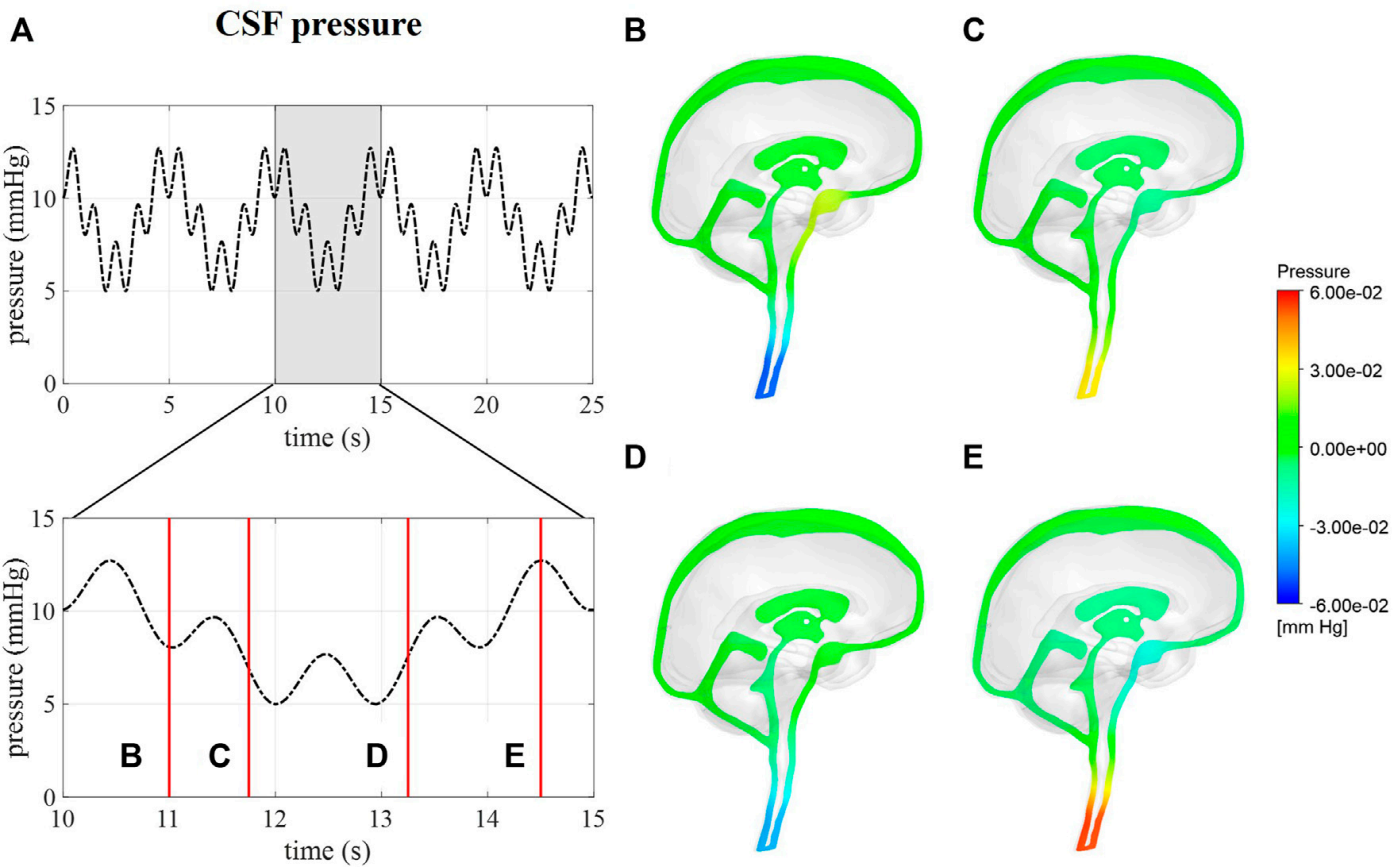
CFD pressure for case **(B)** A pressure averaged over the interstitial outlet over 25 s and then zoomed in on 5 s. **(B–E)** pressure contours at 11, 11.75, 13.25, and 14.5 s, respectively.

Intracranial pressure, corresponding with average pressure at the interstitial outlet (int), is presented over 25 s (Figure 6). Contours are visualized for four different timepoints along one respiratory cycle showing the spatial pressure differences with the pressure at the interstitial outlet (int) as reference. These spatial pressure differences are much smaller than the temporal pressure differences, which corresponds to the low CSF flow. Fluctuations of CSF flow led to spatial fluctuations of pressure in the spinal SAS following CSF pulsations.

**FIGURE 7:**
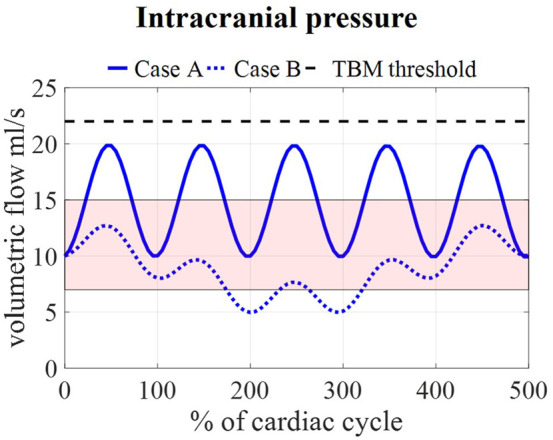
Model pressure for cases A and B is visualized together with the physiological range of 7–15 mmHg reported in (Eide and Kerty, 2011; Dai et al., 2020; Singh and Cheng, 2021) and the threshold for maximal pressure following the guidelines for traumatic brain injury (TBM) (Carney et al., 2017) for 5 cardiac cycles.

Calculated pressures for cases A and B are visualized in Figure 7 for one cardiac cycle together with the average physiological range of 7–15 mmHg (Eide and Kerty, 2011; Dai et al., 2020; Singh and Cheng, 2021). Also, the absolute threshold of 22 mmHg for intracranial pressure (recommended for management of traumatic brain injury (Carney et al., 2017; Hawryluk et al., 2020)) is added. The average pressure over 25 cardiac cycles is 14.8 mmHg for case A and 8.9 mmHg for cases B, C, and D compared to 14.9 and 7.7 predicted using the 0D model.

### 3.4 Impact of compliance magnitude and distribution (case B–E)

#### 3.4.1 CSF flows and pressures

**FIGURE 8:**
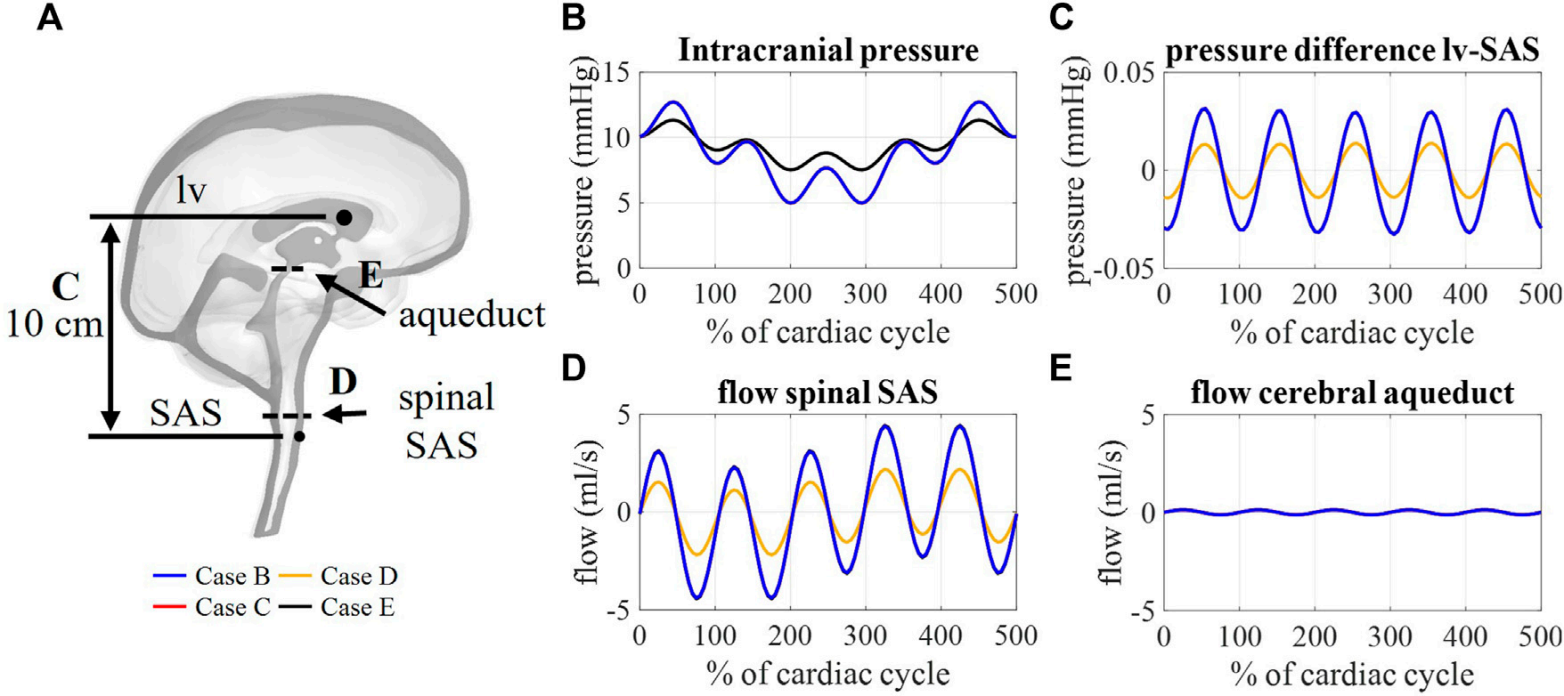
**(A)** Sagittal cross-section CSF depicting the location of aqueduct cross-section B, spinal SAS cross-section D, and 10 cm distance between a point in lateral ventricles (lv) and spinal SAS (SAS) **(C)**. Simulation results for cases B, C, D, and E: **(B)** Intracranial pressure corresponding to interstitial outlet (int), **(C)** spatial pressure difference between a point in lateral ventricle and spinal SAS, **(D)** and **(E)** flow through a cross-section of the spinal SAS and cerebral aqueduct, respectively.

The flow through the cerebral aqueduct (Figure 8E) is identical for all simulations and does not change with compliance. Flow at the spinal SAS level (Figure 8D), however, is dependent on compliance distribution with case D (reduced contribution of spinal compliance) leading to reduced flow pulsations. Figure 8C presents the spatial difference in pressure between a point in the lateral ventricles and a point in the upper part of the spinal SAS. This pressure difference is only impacted by changes in the spinal compliance contribution. Here, an amplitude of maximal 0.03 mmHg is predicted for cases B, C, and E against 0.015 mmHg for case D. In contrast, doubling the intracranial compliance between B and E resulted in a reduction of average intracranial pressure as presented in Figure 8B.

#### 3.4.2 CSF outflows

**FIGURE 9:**
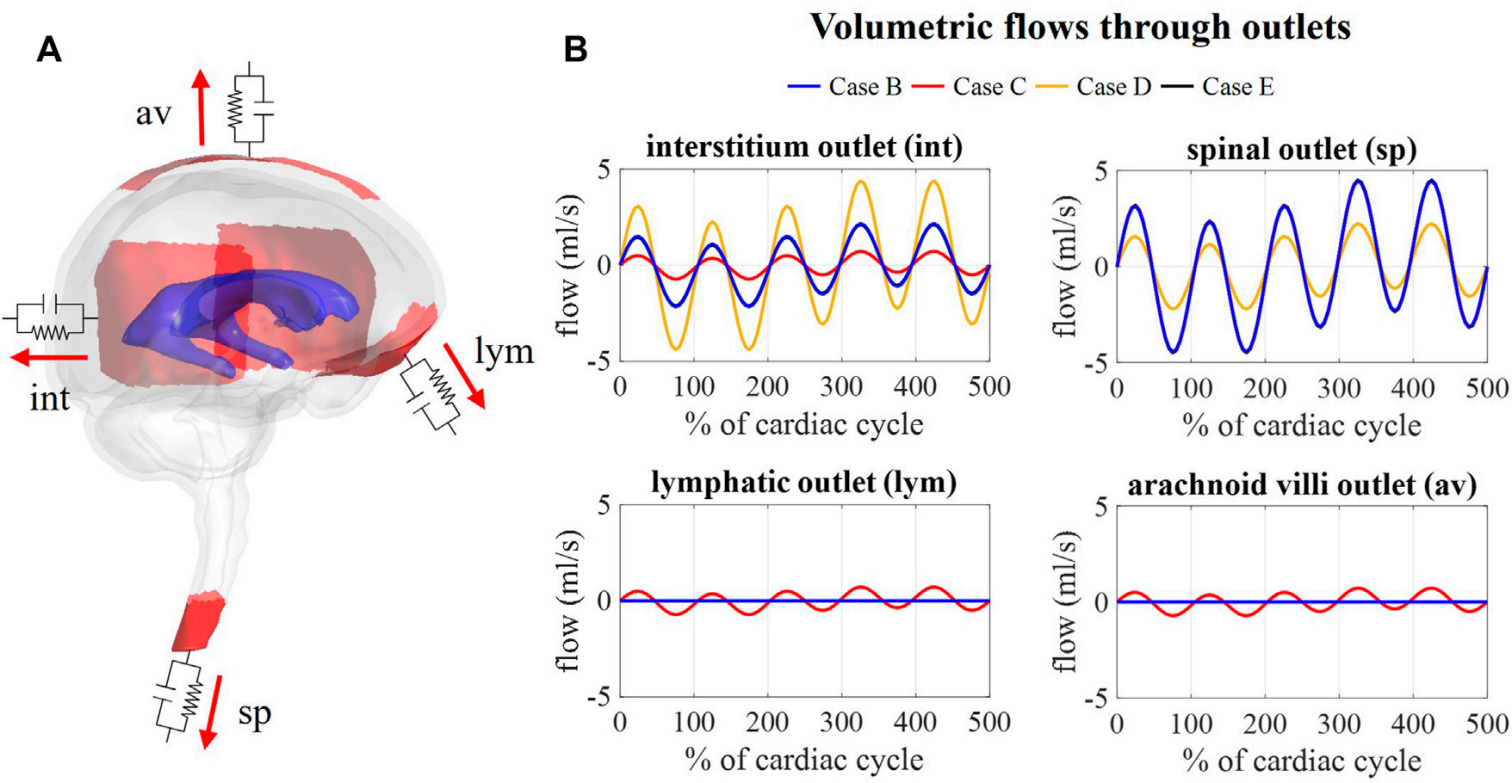
Flow obtained at each outlet for different values of compliance corresponding. **(A)** Visualization of outlets with windkessel boundary conditions. **(B)** Graphs showing the flow through the interstitium, spinal, lymphatic, and arachnoid villi outlet for case B, C, D, and (E).

Figure 9B depicts the outflows for the three intracranial (int, lym, and av) and the spinal (sp) outlets. For case B, arterial and venous volume changes are accommodated by the spinal and interstitial outlet, whereas a flow following the pressure fluctuations is observed for the lymphatic and arachnoid villi outlets. Redistributing the compliance over all outlets in case C results in a pulsatile flow through all outlets. Further, the amplitude of the flow through the spinal and interstitial outlet is switched between cases B and D, where the spinal and interstitium compliance contribution are changed as summarized in Table 3. Finally, no difference in outflow is predicted between cases B and E.

## 4 Discussion

A computational fluid dynamics model of the CSF is presented where fluid pressures and flow are reproduced through the implementation of physiological boundary conditions. First, a 0D model was used to define an adequate value of total compliance by simulating pressure for different compliance values and then selecting the value yielding physiological pressure pulsations. Different compliance values were obtained for case A without and case B with respiration effects. Interestingly, it is only when considering the effects of respiration that we obtained a compliance value within the range derived from *in vivo* measurements (0.4–1.2 ml/mmHg) (Magnaes, 1989; Portella et al., 2005; Tain et al., 2011; Eide, 2016), suggesting that discarding respiration leads to an underestimation of the intracranial compliance. The amplitude of pressure pulsations predicted by the 3D model is 98% and 78% of the amplitude obtained for the 0D model for cases A and B, respectively. This difference in pressure can be attributed to the simplifications in the 0D model of one inlet combining all inflow boundary conditions compared to the spatial distribution of the flow in the 3D model.

Since MRI data reported in previous studies are typically obtained through cardiac gated MRI acquisition, only simulation results without respiratory influences were compared to *in vivo* obtained flow values. Here, fluid flow across a cross-section of the SAS and the cerebral aqueduct showed a good agreement with phase-contrast MRI measurements (Figure 4) with aqueduct flow in the order of 0.11 ml/s and SAS flow in the order of 3.4 ml/s. This model, thereby, produces similar flow amplitudes as obtained in previous studies for the third ventricle and aqueduct in (Sweetman et al., 2011; Wagshul et al., 2011) and the SAS in CFD (Fillingham et al., 2022; Khani et al., 2022) and *in vitro* studies (Benninghaus et al., 2019). Adding respiration resulted in a pulsatile flow pattern across the foramen magnum with, evidently, the appearance of pulsations at the imposed frequencies (Figure 5) of 1 (cardiac) and 0.2 Hz (respiration). Maximal velocities at the cerebral aqueduct are 1.2 cm/s, which is in the same order of magnitude as velocity measurements reported in (Matsumae et al., 2019). However, these velocities are lower than those presented in computational studies with maximal velocities of 2.4 cm/s (Sweetman et al., 2011) and 2 cm/s (Fillingham et al., 2022) in the cerebral aqueduct, and *in vivo* measurements of 3–4 cm/s reported for a reference cohort in (Eide et al., 2021).

We assumed that the average pressure is determined by the absorption resistance and production rate. However, in the simulations, time-average pressures of 14.8 mmHg for case A and 8.9 mmHg for case B were obtained at the interstitial outlet (int). This is because the simulations were only run for 25 cardiac cycles to save computational time. The 0D results, presented in Figure 3, showed that for both cases A and B average pressure evolves toward a steady state after only 2,000 cardiac cycles yielding an average of 10 mmHg, the targeted average intracranial pressure. It is, however, not feasible to do these long simulations using the 3D CFD model. Nevertheless, CFD average pressures lay within the physiological range (Figure 7), which is typically considered 7–15 mmHg (Eide and Kerty, 2011; Dai et al., 2020; Singh and Cheng, 2021), and below the absolute threshold of 22 mmHg recommended for management of traumatic brain injury in 2017. This value was selected because a significant rise in mortality of traumatic brain injury patients was observed when an intracranial pressure higher than 22 mmHg was sustained for 5 days (Carney et al., 2017).

The possibility to obtain absolute CSF pressures and velocities simultaneously (Figures 5, 6) is an important advantage of this model over previous CFD models that do not provide absolute intracranial pressures (Khani et al., 2020; Fillingham et al., 2022). Meanwhile, the FSI model by Sweetman et al. (2011) (Sweetman et al., 2011) did provide intracranial pressure over time with average pressures of about 575 Pa (4.3 mmHg) and a maximal pressure difference of 175 Pa (1.3 mmHg). They predicted maximal spatial pressure differences of 0.2 mmHg between the lateral ventricles and the cervical spinal SAS, which is nearly 10 times larger than the pressure difference of 0.03 mmHg obtained for case B in our model. In contrast, the CFD model by Fillingham et al. (2022) reported maximal pressure differences of 7 Pa or 0.05 mmHg over the cranial CSF space. *In vivo* pressure measurements in patients with idiopathic normal pressure hydrocephalus suggest a maximal pressure difference between the subdural and ventricles of 1 mmHg/m or 0.1 mmHg difference between the ventricles and foramen magnum (10 cm) (Vinje et al., 2019). Thus, both our model and Fillingham et al. (2022) CFD model seem to underpredict, while the FSI model by Sweetman et al. overpredicts the spatial pressure differences. The lower pressure difference in our model might be attributed to the larger diameter of the cerebral aqueduct in our model compared to the physiological diameter. Also, it should be noted that pressure differences in hydrocephalus patients might differ from normal physiological conditions.

Multiple windkessel outlets enable modeling a heterogenous CSF compliance and absorption, which are thought to be impaired in different types of hydrocephalus (Egnor, 2004; Bateman and Siddique, 2014). The inclusion of resistances allows us to easily steer absorption through four different outlets, thereby, no longer ignoring system-wide absorption in both the lymphatic and venous systems. To the best of our knowledge, this is the first 3D computational study to include both venous and lymphatic absorption, and a heterogeneous compliance distribution over the spinal and intracranial compartment. The total CSF compliance was found to impact the overall CSF pressures, whereby an increase in total compliance reduced the amplitude of pressure pulsations (Figure 8B). Moreover, simulation outcomes indicate that the location and distribution of compliance impact CSF flow. The amplitude of flow through each outlet was proportional to the contribution of that outlet to the total compliance. Consequently, the distribution of compliance could impact the flow through the spinal SAS as observed in Figure 8D between cases B and D (switching the spinal and intracranial compliance). These results suggest that adequate distribution of compliance is important to predict physiological CSF flows.

### 4.1 Limitations and future perspectives

First, the simulation results are validated by comparison to literature data, which are recorded at a limited number of points or planes in the CSF space. Also, these are typically obtained through cardiac-gated acquisition only, meaning that the measurements are not real-time and respiration information is not quantified. In that way, they do not account for respiratory effects. Therefore, only simulation results without respiratory influences (case A) were compared to flow measurements. Pressure recordings presented are typically acquired in the context of traumatic brain injury (Carney et al., 2017; Dai et al., 2020) or hydrocephalus (Eide, 2016) and may not reflect normal physiological pressures. Recordings in subjects with physiological intracranial pressures would allow for expanding our validation. The parameters in our model including inlet boundary conditions, resistance, and compliance, can easily be adapted once more measurement data becomes available. Our data indicate that the boundary conditions can be optimized to simultaneously obtain pressure and velocities in the physiological range.

Further, this model includes some important simplifications. First, the currently used 3D geometry is simplified to overcome the limited resolution of the original scan (slice thickness of 3 mm) and reduce complexity to limit computer time. Second, the model geometry was obtained from clinical MRI images of a patient with Chiari type 1 malformation. However, above mentioned simplifications of the CSF geometry resulted in a CSF layer at the level of the foramen magnum of minimally 4 mm, hereby removing the obstruction from the segmented geometry. As such, simulations are representative for a healthy subject, rather than for a patient with Chiari malformation. Third, the locations of the veins and arteries are limited to two locations and the morphology of the boundary surfaces is loosely based on literature. Fourth, the inlet boundary conditions are simplified to sinusoidal time signals with a constant value over the complete inlet surface. Last, CSF volume compensation mechanisms within and beyond the simulated 3D space are simplified by introducing windkessel models including compliance at the outlets. These models are distributed, yet still implemented at a discrete and limited number of sites. It is, without any doubt, more physiologically correct to use an FSI approach to account for compliance effects arising from the deformability of the tissues surrounding the simulated 3D fluid spaces, as demonstrated by among others (Gholampour and Fatouraee, 2021). As such, although FSI models are computationally more complex and require adequate material models and properties for brain tissues, future research should aim to include FSI to achieve more physiological fluid displacements across the simulated 3D space.

Despite these simplifications and assumptions, this model is a proof of concept that by setting proper boundary conditions, thus inlet and windkessel boundary conditions in a 3D model of the CSF, one can obtain pressures and velocities within the physiological range. Now, the model can be stepwise optimized, including more detailed information on the neural anatomy and physiology. Toward the future, we aim to apply this framework to neurological disorders with known disruptions of CSF pressure and flow and to expand validation to extended datasets, including data on the cerebral arteries and veins.

## 5 Conclusion

A computational model of the 3D CSF space has been developed. Through the implementation of physiological processes and adequate tuning of inlet- and outlet boundary conditions, the model yielded CSF pressures and velocities within the physiological range as indicated by invasive intracranial pressure monitoring and phase-contrast MRI. CSF absorption and compliance are modeled by the implementation of 2-element windkessel models at the outlets. We found that the distribution of compliance has an important impact on CSF flow direction, while total compliance impacts the amplitude of the pressure fluctuations.

## Data Availability

The raw data supporting the conclusion of this article will be made available by the authors, without undue reservation.

## Abbreviations

CSF, cerebrospinal fluid; SAS, subarachnoid space; MRI, magnetic resonance imaging; CFD, computational fluid dynamics; TBM, traumatic brain injury; ICP, intracranial pressure; FSI, fluid-structure interaction; int, interstitial outlet; sp, spinal outlet; lym, lymphatic outlet; av, arachnoid villi outlet.
