# Supplementary figures and images for: Computational fluid dynamics model to predict the dynamical behavior of the cerebrospinal fluid through implementation of physiological boundary conditions
Source: Front Bioeng Biotechnol. 2022 Nov 22;10:1040517. doi: 10.3389/fbioe.2022.1040517 (PMC9722737; doi:10.3389/fbioe.2022.1040517)

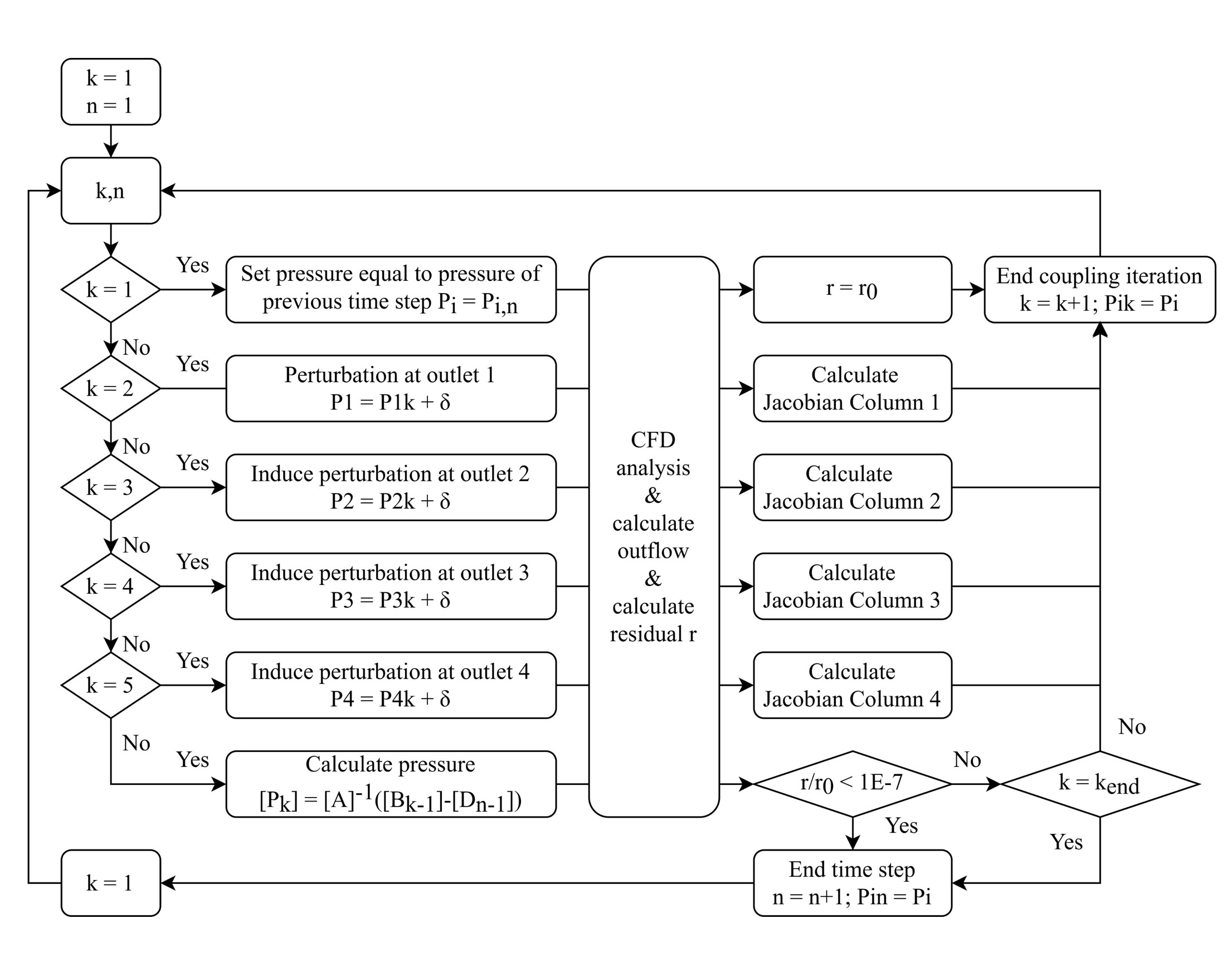

Supplement: Supplementary file 1 [file Image1.JPEG]
