# Supplementary material for: Computational fluid dynamics model to predict the dynamical behavior of the cerebrospinal fluid through implementation of physiological boundary conditions
Source: Front Bioeng Biotechnol. 2022 Nov 22;10:1040517. doi: 10.3389/fbioe.2022.1040517 (PMC9722737; doi:10.3389/fbioe.2022.1040517)
Supplement: Supplementary file 2 [file DataSheet1.docx]

Supplementary Material

# Details on coupling algorithm for implementation in CFD solver

In this section, more details on the implementation of the coupling algorithm for implementation of windkessel boundary conditions are provided. This approach is based on the coupling algorithm described in (Annerel et al., 2012, Annerel et al., 2010). The overview of the coupling scheme is shown in Supplementary Figure 1. First, an explicit expression is derived for pressure (P_i,n_) at outlet i in function of the outflow flow at timestep n (Q_n_) and the pressure in the previous timestep n-1 as discussed in section 2.3.2.1 of the article.

$$\boldsymbol{P}_{\boldsymbol{i}\boldsymbol{,}\boldsymbol{n}}\mathbf{=}\frac{\boldsymbol{Q}_{\boldsymbol{i}\boldsymbol{,}\boldsymbol{n}}\boldsymbol{R}_{\boldsymbol{i}}\mathbf{+}\boldsymbol{P}_{\boldsymbol{i}\boldsymbol{,}\boldsymbol{n}\boldsymbol{-}\boldsymbol{1}}\frac{\boldsymbol{C}_{\boldsymbol{i}}\boldsymbol{R}_{\boldsymbol{i}}}{\boldsymbol{\Delta}\boldsymbol{t}}}{\boldsymbol{1}\boldsymbol{+}\frac{\boldsymbol{C}_{\boldsymbol{i}}\boldsymbol{R}_{\boldsymbol{i}}}{\boldsymbol{\Delta}\boldsymbol{t}}}\boldsymbol{(}\boldsymbol{1}\boldsymbol{)}$$

Each timestep is subdivided into couplings iterations and the windkessel formulation between pressure and flow is linearized for coupling iteration k and time step n.

$$\left\{ \begin{matrix} \begin{matrix} \boldsymbol{Q}_{\boldsymbol{1}\boldsymbol{,}\boldsymbol{k}}\boldsymbol{=}\boldsymbol{Q}_{\boldsymbol{1}\boldsymbol{,}\boldsymbol{k}\boldsymbol{-}\boldsymbol{1}}\boldsymbol{+}\sum_{\boldsymbol{i}\boldsymbol{=}\boldsymbol{1}}^{\boldsymbol{4}} \left. \frac{\boldsymbol{\partial}\boldsymbol{Q}_{\boldsymbol{1}}}{\boldsymbol{\partial}\boldsymbol{P}_{\boldsymbol{i}}} \right|_{\boldsymbol{k}}\left( \boldsymbol{p}_{\boldsymbol{i}\boldsymbol{,}\boldsymbol{k}}\boldsymbol{-}\boldsymbol{p}_{\boldsymbol{i}\boldsymbol{,}\boldsymbol{k}\boldsymbol{-}\boldsymbol{1}} \right) \\ \boldsymbol{Q}_{\boldsymbol{2}\boldsymbol{,}\boldsymbol{k}}\boldsymbol{=}\boldsymbol{Q}_{\boldsymbol{2}\boldsymbol{,}\boldsymbol{k}\boldsymbol{-}\boldsymbol{1}}\boldsymbol{+}\sum_{\boldsymbol{i}\boldsymbol{=}\boldsymbol{1}}^{\boldsymbol{4}} \left. \frac{\boldsymbol{\partial}\boldsymbol{Q}_{\boldsymbol{2}}}{\boldsymbol{\partial}\boldsymbol{P}_{\boldsymbol{i}}} \right|_{\boldsymbol{k}}\left( \boldsymbol{p}_{\boldsymbol{i}\boldsymbol{,}\boldsymbol{k}}\boldsymbol{-}\boldsymbol{p}_{\boldsymbol{i}\boldsymbol{,}\boldsymbol{k}\boldsymbol{-}\boldsymbol{1}} \right) \end{matrix} \\ \begin{matrix} \boldsymbol{Q}_{\boldsymbol{3}\boldsymbol{,}\boldsymbol{k}}\boldsymbol{=}\boldsymbol{Q}_{\boldsymbol{3}\boldsymbol{,}\boldsymbol{k}\boldsymbol{-}\boldsymbol{1}}\boldsymbol{+}\sum_{\boldsymbol{i}\boldsymbol{=}\boldsymbol{1}}^{\boldsymbol{4}} \left. \frac{\boldsymbol{\partial}\boldsymbol{Q}_{\boldsymbol{3}}}{\boldsymbol{\partial}\boldsymbol{P}_{\boldsymbol{i}}} \right|_{\boldsymbol{k}}\left( \boldsymbol{p}_{\boldsymbol{i}\boldsymbol{,}\boldsymbol{k}}\boldsymbol{-}\boldsymbol{p}_{\boldsymbol{i}\boldsymbol{,}\boldsymbol{k}\boldsymbol{-}\boldsymbol{1}} \right) \\ \boldsymbol{Q}_{\boldsymbol{4}\boldsymbol{,}\boldsymbol{k}}\boldsymbol{=}\boldsymbol{Q}_{\boldsymbol{4}\boldsymbol{,}\boldsymbol{k}\boldsymbol{-}\boldsymbol{1}}\boldsymbol{+}\sum_{\boldsymbol{i}\boldsymbol{=}\boldsymbol{1}}^{\boldsymbol{4}} \left. \frac{\boldsymbol{\partial}\boldsymbol{Q}_{\boldsymbol{4}}}{\boldsymbol{\partial}\boldsymbol{P}_{\boldsymbol{i}}} \right|_{\boldsymbol{k}}\left( \boldsymbol{p}_{\boldsymbol{i}\boldsymbol{,}\boldsymbol{k}}\boldsymbol{-}\boldsymbol{p}_{\boldsymbol{i}\boldsymbol{,}\boldsymbol{k}\boldsymbol{-}\boldsymbol{1}} \right) \end{matrix} \end{matrix} \right.\boldsymbol{(}\boldsymbol{2}\boldsymbol{)}$$

Subsequently, the linearized equations in (2) are substituted in the windkessel equation (1) resulting in the following set of equations.

$$\left\{ \begin{matrix} \begin{matrix} \boldsymbol{P}_{\boldsymbol{1}\boldsymbol{,}\boldsymbol{k}}\left( \frac{\boldsymbol{1}}{\boldsymbol{R}_{\boldsymbol{1}}}\boldsymbol{+}\frac{\boldsymbol{C}_{\boldsymbol{1}}}{\boldsymbol{\Delta}\boldsymbol{t}} \right)\boldsymbol{-}\sum_{\boldsymbol{i}\boldsymbol{=}\boldsymbol{1}}^{\boldsymbol{4}} \left. \frac{\boldsymbol{\partial}\boldsymbol{Q}_{\boldsymbol{1}}}{\boldsymbol{\partial}\boldsymbol{P}_{\boldsymbol{i}}} \right|_{\boldsymbol{k}}\boldsymbol{P}_{\boldsymbol{i}\boldsymbol{,}\boldsymbol{k}}\boldsymbol{=}\boldsymbol{Q}_{\boldsymbol{1}\boldsymbol{,}\boldsymbol{k}\boldsymbol{-}\boldsymbol{1}}\boldsymbol{-}\boldsymbol{P}_{\boldsymbol{1}\boldsymbol{,}\boldsymbol{n}\boldsymbol{-}\boldsymbol{1}}\frac{\boldsymbol{C}_{\boldsymbol{1}}}{\boldsymbol{\Delta}\boldsymbol{t}}\boldsymbol{-}\sum_{\boldsymbol{i}\boldsymbol{=}\boldsymbol{1}}^{\boldsymbol{4}} \left. \frac{\boldsymbol{\partial}\boldsymbol{Q}_{\boldsymbol{1}}}{\boldsymbol{\partial}\boldsymbol{P}_{\boldsymbol{i}}} \right|_{\boldsymbol{k}}\boldsymbol{P}_{\boldsymbol{i}\boldsymbol{,}\boldsymbol{k}\boldsymbol{-}\boldsymbol{1}} \\ \boldsymbol{P}_{\boldsymbol{2}\boldsymbol{,}\boldsymbol{k}}\left( \frac{\boldsymbol{1}}{\boldsymbol{R}_{\boldsymbol{2}}}\boldsymbol{+}\frac{\boldsymbol{C}_{\boldsymbol{2}}}{\boldsymbol{\Delta}\boldsymbol{t}} \right)\boldsymbol{-}\sum_{\boldsymbol{i}\boldsymbol{=}\boldsymbol{1}}^{\boldsymbol{4}} \left. \frac{\boldsymbol{\partial}\boldsymbol{Q}_{\boldsymbol{2}}}{\boldsymbol{\partial}\boldsymbol{P}_{\boldsymbol{i}}} \right|_{\boldsymbol{k}}\boldsymbol{P}_{\boldsymbol{i}\boldsymbol{,}\boldsymbol{k}}\boldsymbol{=}\boldsymbol{Q}_{\boldsymbol{2}\boldsymbol{,}\boldsymbol{k}\boldsymbol{-}\boldsymbol{1}}\boldsymbol{-}\boldsymbol{P}_{\boldsymbol{2}\boldsymbol{,}\boldsymbol{n}\boldsymbol{-}\boldsymbol{1}}\frac{\boldsymbol{C}_{\boldsymbol{2}}}{\boldsymbol{\Delta}\boldsymbol{t}}\boldsymbol{-}\sum_{\boldsymbol{i}\boldsymbol{=}\boldsymbol{1}}^{\boldsymbol{4}} \left. \frac{\boldsymbol{\partial}\boldsymbol{Q}_{\boldsymbol{2}}}{\boldsymbol{\partial}\boldsymbol{P}_{\boldsymbol{i}}} \right|_{\boldsymbol{k}}\boldsymbol{P}_{\boldsymbol{i}\boldsymbol{,}\boldsymbol{k}\boldsymbol{-}\boldsymbol{1}} \end{matrix} \\ \begin{matrix} \boldsymbol{P}_{\boldsymbol{3}\boldsymbol{,}\boldsymbol{k}}\left( \frac{\boldsymbol{1}}{\boldsymbol{R}_{\boldsymbol{3}}}\boldsymbol{+}\frac{\boldsymbol{C}_{\boldsymbol{3}}}{\boldsymbol{\Delta}\boldsymbol{t}} \right)\boldsymbol{-}\sum_{\boldsymbol{i}\boldsymbol{=}\boldsymbol{1}}^{\boldsymbol{4}} \left. \frac{\boldsymbol{\partial}\boldsymbol{Q}_{\boldsymbol{3}}}{\boldsymbol{\partial}\boldsymbol{P}_{\boldsymbol{i}}} \right|_{\boldsymbol{k}}\boldsymbol{P}_{\boldsymbol{i}\boldsymbol{,}\boldsymbol{k}}\boldsymbol{=}\boldsymbol{Q}_{\boldsymbol{3}\boldsymbol{,}\boldsymbol{k}\boldsymbol{-}\boldsymbol{1}}\boldsymbol{-}\boldsymbol{P}_{\boldsymbol{3}\boldsymbol{,}\boldsymbol{n}\boldsymbol{-}\boldsymbol{1}}\frac{\boldsymbol{C}_{\boldsymbol{3}}}{\boldsymbol{\Delta}\boldsymbol{t}}\boldsymbol{-}\sum_{\boldsymbol{i}\boldsymbol{=}\boldsymbol{1}}^{\boldsymbol{4}} \left. \frac{\boldsymbol{\partial}\boldsymbol{Q}_{\boldsymbol{3}}}{\boldsymbol{\partial}\boldsymbol{P}_{\boldsymbol{i}}} \right|_{\boldsymbol{k}}\boldsymbol{P}_{\boldsymbol{i}\boldsymbol{,}\boldsymbol{k}\boldsymbol{-}\boldsymbol{1}} \\ \boldsymbol{P}_{\boldsymbol{4}\boldsymbol{,}\boldsymbol{k}}\left( \frac{\boldsymbol{1}}{\boldsymbol{R}_{\boldsymbol{4}}}\boldsymbol{+}\frac{\boldsymbol{C}_{\boldsymbol{4}}}{\boldsymbol{\Delta}\boldsymbol{t}} \right)\boldsymbol{-}\sum_{\boldsymbol{i}\boldsymbol{=}\boldsymbol{1}}^{\boldsymbol{4}} \left. \frac{\boldsymbol{\partial}\boldsymbol{Q}_{\boldsymbol{4}}}{\boldsymbol{\partial}\boldsymbol{P}_{\boldsymbol{i}}} \right|_{\boldsymbol{k}}\boldsymbol{P}_{\boldsymbol{i}\boldsymbol{,}\boldsymbol{k}}\boldsymbol{=}\boldsymbol{Q}_{\boldsymbol{4}\boldsymbol{,}\boldsymbol{k}\boldsymbol{-}\boldsymbol{1}}\boldsymbol{-}\boldsymbol{P}_{\boldsymbol{4}\boldsymbol{,}\boldsymbol{n}\boldsymbol{-}\boldsymbol{1}}\frac{\boldsymbol{C}_{\boldsymbol{4}}}{\boldsymbol{\Delta}\boldsymbol{t}}\boldsymbol{-}\sum_{\boldsymbol{i}\boldsymbol{=}\boldsymbol{1}}^{\boldsymbol{4}} \left. \frac{\boldsymbol{\partial}\boldsymbol{Q}_{\boldsymbol{4}}}{\boldsymbol{\partial}\boldsymbol{P}_{\boldsymbol{i}}} \right|_{\boldsymbol{k}}\boldsymbol{P}_{\boldsymbol{i}\boldsymbol{,}\boldsymbol{k}\boldsymbol{-}\boldsymbol{1}} \end{matrix} \end{matrix} \right.\boldsymbol{(}\boldsymbol{3}\boldsymbol{)}$$

These equations are rewritten in matrix form.

$$\left( \left[ \boldsymbol{R} \right]\boldsymbol{+}\frac{\boldsymbol{1}}{\boldsymbol{\Delta}\boldsymbol{t}}\left[ \boldsymbol{C} \right]\boldsymbol{-}\left[ \boldsymbol{J} \right] \right)\left[ \boldsymbol{P}_{\boldsymbol{k}} \right]\boldsymbol{=}\left[ \boldsymbol{Q}_{\boldsymbol{k}\boldsymbol{-}\boldsymbol{1}} \right]\boldsymbol{-}\frac{\boldsymbol{1}}{\boldsymbol{\Delta}\boldsymbol{t}}\left[ \boldsymbol{C} \right]\left[ \boldsymbol{P}_{\boldsymbol{n}\boldsymbol{-}\boldsymbol{1}} \right]\boldsymbol{-}\left[ \boldsymbol{J} \right]\left[ \boldsymbol{P}_{\boldsymbol{k}\boldsymbol{-}\boldsymbol{1}} \right]\boldsymbol{(}\boldsymbol{4}\boldsymbol{)}$$

With Jacobian [J], resistance [R], and compliance matrix [C]

$$\left[ \boldsymbol{J} \right]\boldsymbol{=}\left( \begin{matrix} \begin{matrix} \left. \frac{\boldsymbol{\partial}\boldsymbol{Q}_{\boldsymbol{1}}}{\boldsymbol{\partial}\boldsymbol{P}_{\boldsymbol{1}}} \right|_{\boldsymbol{k}} & \left. \frac{\boldsymbol{\partial}\boldsymbol{Q}_{\boldsymbol{1}}}{\boldsymbol{\partial}\boldsymbol{P}_{\boldsymbol{2}}} \right|_{\boldsymbol{k}} \\ \left. \frac{\boldsymbol{\partial}\boldsymbol{Q}_{\boldsymbol{2}}}{\boldsymbol{\partial}\boldsymbol{P}_{\boldsymbol{1}}} \right|_{\boldsymbol{k}} & \left. \frac{\boldsymbol{\partial}\boldsymbol{Q}_{\boldsymbol{2}}}{\boldsymbol{\partial}\boldsymbol{P}_{\boldsymbol{2}}} \right|_{\boldsymbol{k}} \end{matrix} & \begin{matrix} \left. \frac{\boldsymbol{\partial}\boldsymbol{Q}_{\boldsymbol{1}}}{\boldsymbol{\partial}\boldsymbol{P}_{\boldsymbol{3}}} \right|_{\boldsymbol{k}} & \left. \frac{\boldsymbol{\partial}\boldsymbol{Q}_{\boldsymbol{1}}}{\boldsymbol{\partial}\boldsymbol{P}_{\boldsymbol{4}}} \right|_{\boldsymbol{k}} \\ \left. \frac{\boldsymbol{\partial}\boldsymbol{Q}_{\boldsymbol{2}}}{\boldsymbol{\partial}\boldsymbol{P}_{\boldsymbol{3}}} \right|_{\boldsymbol{k}} & \left. \frac{\boldsymbol{\partial}\boldsymbol{Q}_{\boldsymbol{2}}}{\boldsymbol{\partial}\boldsymbol{P}_{\boldsymbol{4}}} \right|_{\boldsymbol{k}} \end{matrix} \\ \begin{matrix} \left. \frac{\boldsymbol{\partial}\boldsymbol{Q}_{\boldsymbol{3}}}{\boldsymbol{\partial}\boldsymbol{P}_{\boldsymbol{1}}} \right|_{\boldsymbol{k}} & \left. \frac{\boldsymbol{\partial}\boldsymbol{Q}_{\boldsymbol{3}}}{\boldsymbol{\partial}\boldsymbol{P}_{\boldsymbol{2}}} \right|_{\boldsymbol{k}} \\ \left. \frac{\boldsymbol{\partial}\boldsymbol{Q}_{\boldsymbol{4}}}{\boldsymbol{\partial}\boldsymbol{P}_{\boldsymbol{1}}} \right|_{\boldsymbol{k}} & \left. \frac{\boldsymbol{\partial}\boldsymbol{Q}_{\boldsymbol{4}}}{\boldsymbol{\partial}\boldsymbol{P}_{\boldsymbol{2}}} \right|_{\boldsymbol{k}} \end{matrix} & \begin{matrix} \left. \frac{\boldsymbol{\partial}\boldsymbol{Q}_{\boldsymbol{3}}}{\boldsymbol{\partial}\boldsymbol{P}_{\boldsymbol{3}}} \right|_{\boldsymbol{k}} & \left. \frac{\boldsymbol{\partial}\boldsymbol{Q}_{\boldsymbol{3}}}{\boldsymbol{\partial}\boldsymbol{P}_{\boldsymbol{4}}} \right|_{\boldsymbol{k}} \\ \left. \frac{\boldsymbol{\partial}\boldsymbol{Q}_{\boldsymbol{4}}}{\boldsymbol{\partial}\boldsymbol{P}_{\boldsymbol{3}}} \right|_{\boldsymbol{k}} & \left. \frac{\boldsymbol{\partial}\boldsymbol{Q}_{\boldsymbol{4}}}{\boldsymbol{\partial}\boldsymbol{P}_{\boldsymbol{4}}} \right|_{\boldsymbol{k}} \end{matrix} \end{matrix} \right)\boldsymbol{;}$$

$$\left[ \boldsymbol{R} \right]\boldsymbol{=}\left( \begin{matrix} \frac{\boldsymbol{1}}{\boldsymbol{R}_{\boldsymbol{1}}} & & \\ & \frac{\boldsymbol{1}}{\boldsymbol{R}_{\boldsymbol{2}}} & \\ & & \begin{matrix} \frac{\boldsymbol{1}}{\boldsymbol{R}_{\boldsymbol{3}}} & \\ & \frac{\boldsymbol{1}}{\boldsymbol{R}_{\boldsymbol{4}}} \end{matrix} \end{matrix} \right)\boldsymbol{;}\left[ \boldsymbol{C} \right]\boldsymbol{=}\left( \begin{matrix} \boldsymbol{C}_{\boldsymbol{1}} & & \\ & \boldsymbol{C}_{\boldsymbol{2}} & \\ & & \begin{matrix} \boldsymbol{C}_{\boldsymbol{3}} & \\ & \boldsymbol{C}_{\boldsymbol{4}} \end{matrix} \end{matrix} \right) \boldsymbol{(}\boldsymbol{5}\boldsymbol{)}$$

Finally, the pressures at the outlets contained in the matrix [P _k+1_] can be written in function of the outlet pressures in previous timestep [P_n-1_], and the pressure [P_k_] and flow [Q_k_] in the previous coupling iteration.

$$\left[ \boldsymbol{P}_{\boldsymbol{k}} \right]\boldsymbol{=}\left( \left[ \boldsymbol{R} \right]\boldsymbol{+}\frac{\boldsymbol{1}}{\boldsymbol{\Delta}\boldsymbol{t}}\left[ \boldsymbol{C} \right]\boldsymbol{-}\left[ \boldsymbol{J} \right] \right)^{\boldsymbol{-}\boldsymbol{1}}\left( \left[ \boldsymbol{Q}_{\boldsymbol{k}\boldsymbol{-}\boldsymbol{1}} \right]\boldsymbol{-}\frac{\boldsymbol{1}}{\boldsymbol{\Delta}\boldsymbol{t}}\left[ \boldsymbol{C} \right]\left[ \boldsymbol{P}_{\boldsymbol{n}\boldsymbol{-}\boldsymbol{1}} \right]\boldsymbol{-}\left[ \boldsymbol{J} \right]\left[ \boldsymbol{P}_{\boldsymbol{k}\boldsymbol{-}\boldsymbol{1}} \right] \right)\boldsymbol{(}\boldsymbol{6}\boldsymbol{)}$$

This can be written in short as

$$\left[ \boldsymbol{P}_{\boldsymbol{k}} \right]\boldsymbol{=}{\boldsymbol{[}\boldsymbol{A}\boldsymbol{]}}^{\boldsymbol{-}\boldsymbol{1}}\left( \left[ \boldsymbol{B}_{\boldsymbol{k}\boldsymbol{-}\boldsymbol{1}} \right]\boldsymbol{-}\left[ \boldsymbol{D}_{\boldsymbol{n}\boldsymbol{-}\boldsymbol{1}} \right] \right)\boldsymbol{(}\boldsymbol{7}\boldsymbol{)}$$

The Jacobian [J] contains the derivatives of flow with respect to pressure. These derivatives are approximated by finite differences. Following Supplementary Figure 1, the first five coupling iterations are used to calculate the Jacobian, whereby a perturbation δ is subsequently added to each outlet (equation 8). The perturbation is set equal to 1E-5 Pa for case A and 2E-5 Pa for cases B, C, D and E.

$$\boldsymbol{k=1}\boldsymbol{\Rightarrow}\left\{ \begin{matrix} \begin{matrix} \boldsymbol{P}_{\boldsymbol{1,1}}\boldsymbol{=}\boldsymbol{P}_{\boldsymbol{1,n-1}} \\ \boldsymbol{P}_{\boldsymbol{2,1}}\boldsymbol{=}\boldsymbol{P}_{\boldsymbol{2,n-1}} \end{matrix} \\ \begin{matrix} \boldsymbol{P}_{\boldsymbol{3,1}}\boldsymbol{=}\boldsymbol{P}_{\boldsymbol{3,n-1}} \\ \boldsymbol{P}_{\boldsymbol{4,1}}\boldsymbol{=}\boldsymbol{P}_{\boldsymbol{4,n-1}} \end{matrix} \end{matrix}\Rightarrow\left\{ \begin{matrix} \begin{matrix} \boldsymbol{Q}_{\boldsymbol{1,1}} \\ \boldsymbol{Q}_{\boldsymbol{2,1}} \end{matrix} \\ \begin{matrix} \boldsymbol{Q}_{\boldsymbol{3,1}} \\ \boldsymbol{Q}_{\boldsymbol{4,1}} \end{matrix} \end{matrix} \right. \right.$$

$$\boldsymbol{k=2}\boldsymbol{\Rightarrow}\left\{ \begin{matrix} \begin{matrix} \boldsymbol{P}_{\boldsymbol{1,2}}\boldsymbol{=}\boldsymbol{P}_{\boldsymbol{1,1}}\boldsymbol{+\delta} \\ \boldsymbol{P}_{\boldsymbol{2,2}}\boldsymbol{=}\boldsymbol{P}_{\boldsymbol{2,1}} \end{matrix} \\ \begin{matrix} \boldsymbol{P}_{\boldsymbol{3,2}}\boldsymbol{=}\boldsymbol{P}_{\boldsymbol{3,1}} \\ \boldsymbol{P}_{\boldsymbol{4,2}}\boldsymbol{=}\boldsymbol{P}_{\boldsymbol{4,1}} \end{matrix} \end{matrix}\Rightarrow\left\{ \begin{matrix} \begin{matrix} \boldsymbol{Q}_{\boldsymbol{1,2}} \\ \boldsymbol{Q}_{\boldsymbol{2,2}} \end{matrix} \\ \begin{matrix} \boldsymbol{Q}_{\boldsymbol{3,2}} \\ \boldsymbol{Q}_{\boldsymbol{4,2}} \end{matrix} \end{matrix} \right. \right.$$

$$\boldsymbol{k=3}\boldsymbol{\Rightarrow}\left\{ \begin{matrix} \begin{matrix} \boldsymbol{P}_{\boldsymbol{1,3}}\boldsymbol{=}\boldsymbol{P}_{\boldsymbol{1,2}} \\ \boldsymbol{P}_{\boldsymbol{2,3}}\boldsymbol{=}\boldsymbol{P}_{\boldsymbol{2,2}}\boldsymbol{+\delta} \end{matrix} \\ \begin{matrix} \boldsymbol{P}_{\boldsymbol{3,3}}\boldsymbol{=}\boldsymbol{P}_{\boldsymbol{3,2}} \\ \boldsymbol{P}_{\boldsymbol{4,3}}\boldsymbol{=}\boldsymbol{P}_{\boldsymbol{4,2}} \end{matrix} \end{matrix}\boldsymbol{\Rightarrow}\left\{ \begin{matrix} \begin{matrix} \boldsymbol{Q}_{\boldsymbol{1,3}} \\ \boldsymbol{Q}_{\boldsymbol{2,3}} \end{matrix} \\ \begin{matrix} \boldsymbol{Q}_{\boldsymbol{3,3}} \\ \boldsymbol{Q}_{\boldsymbol{4,3}} \end{matrix} \end{matrix} \right. \right.\boldsymbol{(8)}$$

$$\boldsymbol{k=4}\boldsymbol{\Rightarrow}\left\{ \begin{matrix} \begin{matrix} \boldsymbol{P}_{\boldsymbol{1,4}}\boldsymbol{=}\boldsymbol{P}_{\boldsymbol{1+3}} \\ \boldsymbol{P}_{\boldsymbol{2,4}}\boldsymbol{=}\boldsymbol{P}_{\boldsymbol{2+3}} \end{matrix} \\ \begin{matrix} \boldsymbol{P}_{\boldsymbol{3,4}}\boldsymbol{=}\boldsymbol{P}_{\boldsymbol{3+3}}\boldsymbol{+\delta} \\ \boldsymbol{P}_{\boldsymbol{4,4}}\boldsymbol{=}\boldsymbol{P}_{\boldsymbol{4,3}} \end{matrix} \end{matrix}\Rightarrow\left\{ \begin{matrix} \begin{matrix} \boldsymbol{Q}_{\boldsymbol{1,4}} \\ \boldsymbol{Q}_{\boldsymbol{2,4}} \end{matrix} \\ \begin{matrix} \boldsymbol{Q}_{\boldsymbol{3,4}} \\ \boldsymbol{Q}_{\boldsymbol{4,4}} \end{matrix} \end{matrix} \right. \right.$$

$$\boldsymbol{k=5}\boldsymbol{\Rightarrow}\left\{ \begin{matrix} \begin{matrix} \boldsymbol{P}_{\boldsymbol{1}\boldsymbol{,}\boldsymbol{5}}\boldsymbol{=}\boldsymbol{P}_{\boldsymbol{1}\boldsymbol{,}\boldsymbol{4}} \\ \boldsymbol{P}_{\boldsymbol{2}\boldsymbol{,}\boldsymbol{5}}\boldsymbol{=}\boldsymbol{P}_{\boldsymbol{2}\boldsymbol{,}\boldsymbol{4}} \end{matrix} \\ \begin{matrix} \boldsymbol{P}_{\boldsymbol{3}\boldsymbol{,}\boldsymbol{5}}\boldsymbol{=}\boldsymbol{P}_{\boldsymbol{3}\boldsymbol{,}\boldsymbol{4}} \\ \boldsymbol{P}_{\boldsymbol{4}\boldsymbol{,}\boldsymbol{5}}\boldsymbol{=}\boldsymbol{P}_{\boldsymbol{4}\boldsymbol{,}\boldsymbol{4}}\boldsymbol{+}\boldsymbol{\delta} \end{matrix} \end{matrix}\boldsymbol{\Rightarrow}\left\{ \begin{matrix} \begin{matrix} \boldsymbol{Q}_{\boldsymbol{1}\boldsymbol{,}\boldsymbol{5}} \\ \boldsymbol{Q}_{\boldsymbol{2}\boldsymbol{,}\boldsymbol{5}} \end{matrix} \\ \begin{matrix} \boldsymbol{Q}_{\boldsymbol{3}\boldsymbol{,}\boldsymbol{5}} \\ \boldsymbol{Q}_{\boldsymbol{4}\boldsymbol{,}\boldsymbol{5}} \end{matrix} \end{matrix} \right. \right.$$

This perturbation impacts the flow Q_i,k+1_ through the different outlets. Finite differences are then used to approximate flow gradients for outlet i = 1-4 and coupling iteration k = 2-5 allowing calculation of the Jacobian.

$$\left. \frac{\boldsymbol{\partial}\boldsymbol{Q}_{\boldsymbol{i}}}{\boldsymbol{\partial}\boldsymbol{P}_{\boldsymbol{j}}} \right|_{\boldsymbol{k}}\boldsymbol{=}\frac{\boldsymbol{Q}_{\boldsymbol{1}\boldsymbol{,}\boldsymbol{k}}\boldsymbol{-}\boldsymbol{Q}_{\boldsymbol{1}\boldsymbol{,}\boldsymbol{k}\boldsymbol{-}\boldsymbol{1}}}{\boldsymbol{P}_{\boldsymbol{1}\boldsymbol{,}\boldsymbol{k}}\boldsymbol{-}\boldsymbol{P}_{\boldsymbol{1}\boldsymbol{,}\boldsymbol{k}\boldsymbol{-}\boldsymbol{1}}}\boldsymbol{=}\frac{\boldsymbol{Q}_{\boldsymbol{1}\boldsymbol{,}\boldsymbol{k}}\boldsymbol{-}\boldsymbol{Q}_{\boldsymbol{1}\boldsymbol{,}\boldsymbol{k}\boldsymbol{-}\boldsymbol{1}}}{\boldsymbol{\delta}}\boldsymbol{(}\boldsymbol{9}\boldsymbol{)}$$

The Jacobian is then applied to derive the pressure following equation 6. Where a Fluent built-in absolute convergence criterion is applied for the solver iterations, a relative convergence criterion is used to check the convergence of each coupling iteration k>1.

$$\frac{\boldsymbol{r}}{\boldsymbol{r}_{\boldsymbol{0}}}\boldsymbol{=}\sqrt{\sum_{\boldsymbol{i}\boldsymbol{=}\boldsymbol{1}}^{\boldsymbol{4}} \left( \boldsymbol{Q}_{\boldsymbol{i}\boldsymbol{,}\boldsymbol{k}}\boldsymbol{-}\boldsymbol{P}_{\boldsymbol{i}\boldsymbol{,}\boldsymbol{k}}\left( \frac{\boldsymbol{1}}{\boldsymbol{R}_{\boldsymbol{i}}}\boldsymbol{+}\frac{\boldsymbol{C}_{\boldsymbol{i}}}{\boldsymbol{\Delta}\boldsymbol{t}} \right)\boldsymbol{+}\boldsymbol{P}_{\boldsymbol{i}\boldsymbol{,}\boldsymbol{n}\boldsymbol{-}\boldsymbol{1}}\frac{\boldsymbol{C}_{\boldsymbol{i}}}{\boldsymbol{\Delta}\boldsymbol{t}} \right)^{\boldsymbol{2}}}\boldsymbol{<}\boldsymbol{1}\boldsymbol{e}\boldsymbol{-}\boldsymbol{7}\boldsymbol{(}\boldsymbol{10}\boldsymbol{)}$$

In this equation, Q_i,k_ and P_i,k_ are the flow and pressure corresponding to coupling iteration k, whereas P_i,n-1_ is the pressure in the previous timestep (n-1). When this condition is fulfilled, the solution advances to the next timestep (n+1) and k is set to 1. When the criterion is not met, a next couplings iteration is started unless the final coupling iteration k_end_ was reached. Where the equations and commands are contained in the UDF file, journal files manage the subsequent actions of applying pressure, solving the Navier-Stokes equation, checking the convergence, and finally printing the results.

# Supplementary Figures

**Supplementary Figure 1.** Schematic overview of coupling algorithm with the four outlets 1, 2, 3 and 4 corresponding to outlet int, sp, lym and av, respectively.

# References

ANNEREL, S., DEGROOTE, J., CLAESSENS, T., DAHL, S. K., SKALLERUD, B., HELLEVIK, L. R., VAN RANSBEECK, P., SEGERS, P., VERDONCK, P. & VIERENDEELS, J. 2012. A fast strong coupling algorithm for the partitioned fluid-structure interaction simulation of BMHVs. *Comput Methods Biomech Biomed Engin,* 15**,** 1281-312.

ANNEREL, S., DEGROOTE, J., CLAESSENS, T. & VIERENDEELS, J. 2010. Evaluation of a new Implicit Coupling Algorithm for the Partitioned Fluid-Structure Interaction Simulation of Bileaflet Mechanical Heart Valves. *IOP Conference Series: Materials Science and Engineering,* 10.
